# Trypanosoma brucei PRMT1 Is a Nucleic Acid Binding Protein with a Role in Energy Metabolism and the Starvation Stress Response

**DOI:** 10.1128/mBio.02430-18

**Published:** 2018-12-18

**Authors:** Lucie Kafková, Chengjian Tu, Kyle L. Pazzo, Kyle P. Smith, Erik W. Debler, Kimberly S. Paul, Jun Qu, Laurie K. Read

**Affiliations:** a Department of Microbiology and Immunology, Jacobs School of Medicine and Biomedical Sciences, University at Buffalo, State University of New York, Buffalo, New York, USA; b Department of Pharmaceutical Sciences, University at Buffalo, Buffalo, New York, USA; c Department of Biological Sciences, Clemson University, Clemson, South Carolina, USA; d Department of Biochemistry and Molecular Biology, Thomas Jefferson University, Philadelphia, Pennsylvania, USA; Harvard T. H. Chan School of Public Health

**Keywords:** PRMT, RNA binding proteins, *Trypanosoma brucei*, arginine methylation, metabolism regulation, stress response

## Abstract

In Trypanosoma brucei and related kinetoplastid parasites, transcription of protein coding genes is largely unregulated. Rather, mRNA binding proteins, which impact processes such as transcript stability and translation efficiency, are the predominant regulators of gene expression. Arginine methylation is a posttranslational modification that preferentially targets RNA binding proteins and is, therefore, likely to have a substantial impact on T. brucei biology. The data presented here demonstrate that cells depleted of T. brucei PRMT1 (*Tb*PRMT1), a major type I protein arginine methyltransferase, exhibit decreased virulence in an animal model. To understand the basis of this phenotype, quantitative global proteomics was employed to measure protein steady-state levels in cells lacking *Tb*PRMT1. The approach revealed striking changes in proteins involved in energy metabolism. Most prominent were a decrease in glycolytic enzyme abundance and an increase in proline degradation pathway components, changes that resemble the metabolic remodeling that occurs during T. brucei life cycle progression. The work describes several RNA binding proteins whose association with mRNA was altered in *Tb*PRMT1-depleted cells, and a large number of *Tb*PRMT1-interacting proteins, thereby highlighting potential *Tb*PRMT1 substrates. Many proteins involved in the T. brucei starvation stress response were found to interact with *Tb*PRMT1, prompting analysis of the response of *Tb*PRMT1-depleted cells to nutrient deprivation. Indeed, depletion of *Tb*PRMT1 strongly hinders the ability of T. brucei to form cytoplasmic mRNA granules under starvation conditions. Finally, this work shows that *Tb*PRMT1 itself binds nucleic acids *in vitro* and *in vivo*, a feature completely novel to protein arginine methyltransferases.

## Abstract (precis)

Trypanosoma brucei infection causes human African trypanosomiasis, also known as sleeping sickness, a disease with a nearly 100% fatality rate when untreated. Current drugs are expensive, toxic, and highly impractical to administer, prompting the community to explore various unique aspects of T. brucei biology in search of better treatments. In this study, we identified the protein arginine methyltransferase (PRMT), *Tb*PRMT1, as a factor that modulates numerous aspects of T. brucei biology. These include glycolysis and life cycle progression signaling, both of which are being intensely researched toward identification of potential drug targets. Our data will aid research in those fields. Furthermore, we demonstrate for the first time a direct association of a PRMT with nucleic acids, a finding we believe could translate to other organisms, including humans, thereby impacting research in fields as distant as human cancer biology and immune response modulation.

## INTRODUCTION

Trypanosoma brucei is a parasitic protozoan causing African sleeping sickness in sub-Saharan Africa. An estimated 70 million people are at risk of the infection, and WHO estimates roughly 20,000 new cases per year when likely underreporting is taken into account (1). Furthermore, animal trypanosomiasis in the African region constitutes a large economic burden. It is estimated that dealing with trypanosomiasis would result in a benefit of approximately 2.5 billion USD to livestock keepers in affected regions over a 20-year period (2). The parasite is transmitted between the mammalian hosts via an insect vector, the tsetse fly (*Glossina* spp.). Throughout its life cycle, T. brucei changes both its morphology and physiology to adjust to nutritional and immunological conditions encountered in the hosts. The bloodstream form (BF) that thrives in mammalian blood utilizes glycolysis, compartmentalized in a specialized organelle called a glycosome, as the main energy source (3). BF cells employ a quorum sensing mechanism to detect a high parasite load and transform to a nondividing stumpy stage that is preadapted to life in the insect vector (4). Once taken up by the fly, parasites progress through the life cycle, further changing their physiology. The procyclic form (PF) inhabiting the fly’s midgut turns to proline degradation coupled to the TCA cycle to cope with the lack of glucose in its environment (5). These changes are reflected in the size of the parasite’s single mitochondrion, which in PF takes up much of the cytoplasmic space, as well as in the utilization of oxidative phosphorylation, which is almost exclusively active in PF. The changes T. brucei undergoes through its life cycle are almost solely controlled at the posttranscriptional level, since T. brucei utilizes polycistronic transcription of functionally unrelated genes and subsequently generates individual mRNAs through the processes of 5′ *trans*-splicing and 3′ cleavage and polyadenylation (6). The major means of gene regulation are embodied in the mRNA binding proteome, which in turn is regulated by a multitude of mechanisms, including posttranslational modifications (PTM). Protein arginine methylation is a PTM that disproportionally targets RNA binding proteins in T. brucei as well as in mammals (7 – 9). Arginine methylation, which in T. brucei affects about 15% of the proteome, is catalyzed by protein arginine methyltransferases (PRMTs) that can be classified into three types based on the end products of their catalytic activities (7, 8, 10). All three types can catalyze the formation of ω-*N^G^*-monomethylarginine (MMA). While type III PRMTs are limited to this modification, type I PRMTs can catalyze formation of ω-*N^G^*,*N^G^*-asymmetric dimethylarginine (ADMA), and type II PRMTs create ω-*N^G^*,*N*′*^G^*-symmetric dimethylarginine (SDMA). T. brucei harbors four PRMTs that represent all three types and engage in a functional interplay (11).

T. brucei PRMT1 (*Tb*PRMT1) is a type I PRMT that we previously showed catalyzes the majority of ADMA formation *in vivo* (12). *Tb*PRMT1 influences the functions of proteins involved in RNA editing and controls the mRNA-stabilizing and -destabilizing functions of the RNA binding protein DRBD18 in PF T. brucei (12 – 16). However, more global impacts on cell function have not been investigated, and *Tb*PRMT1 function has not been examined in BF T. brucei. *Tb*PRMT1 structure is highly unusual since, unlike other characterized type I PRMTs that form homodimers or homomultimers, it functions as a heterotetramer of an enzymatic subunit (ENZ) and a catalytically inactive PRMT paralog termed prozyme (PRO) (13, 17 – 20). The two *Tb*PRMT1 subunits mutually stabilize each other on the protein level, and while the PRO subunit does not carry out catalysis, it is indispensable for *Tb*PRMT1 function.

Here, we examine the *in vivo* role and *in vitro* properties of *Tb*PRMT1 using a mouse model and biochemical, cell biological, and global proteomic strategies in BF and PF T. brucei. Our results show that although *Tb*PRMT1 is not strictly needed for BF T. brucei growth in culture, the protein contributes to T. brucei virulence in an animal model. We further show that in the absence of *Tb*PRMT1, the BF parasites downregulate enzymes involved in glycolysis and upregulate pathways that utilize alternative energy sources. We quantified changes in the mRNA-bound proteome and identified several proteins whose association with mRNA is significantly altered in the *Tb*PRMT1-depleted background. In the attempt to identify *Tb*PRMT1 *in vivo* substrates, we noted an overrepresentation of stress-related proteins associating with *Tb*PRMT1. We confirmed the biological significance of this finding by demonstrating a defect in mRNA granule formation during nutritional stress in PF cells depleted for *Tb*PRMT1. Finally, we show that *Tb*PRMT1 itself is able to associate with nucleic acids, which is a completely novel feature in this class of enzymes. Thus, the present studies reveal that *Tb*PRMT1 plays significant roles in trypanosome virulence, metabolism, and RNA biology.

## RESULTS

### Knockout of the *Tb*PRMT1 enzymatic subunit leads to growth retardation and decreases parasite virulence.

**FIG 1:**
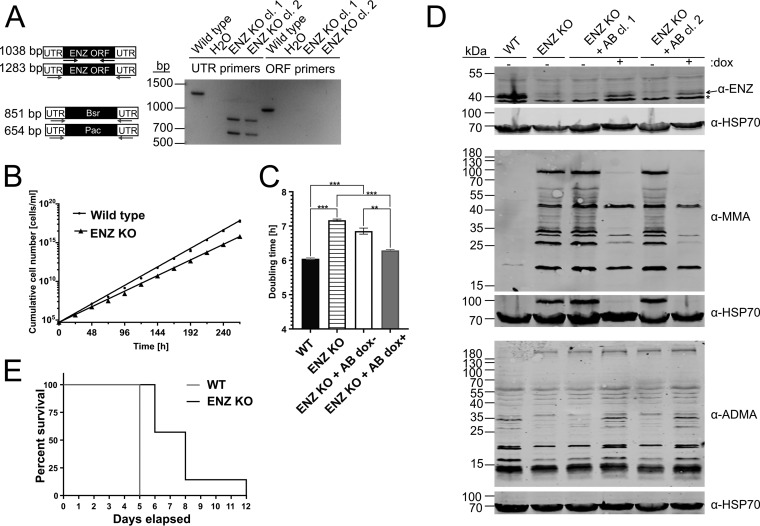
*Tb*PRMT1 contributes to T. brucei virulence. (A) (Left) Schematic representation of the knockout (KO) strategy. Arrows indicate primer placement. (Right) KO confirmation by PCR of genomic DNA from two different clones. (B) Growth of WT and ENZ KO cell lines in culture. Cumulative cell density over time was plotted. Each point represents the mean for two clones in duplicate. Error bars indicate standard deviation. (C) Doubling time of WT, ENZ KO, and ENZ KO+AB (add-back) cells either uninduced (dox-) or induced for expression of exogenous ENZ (dox+) was calculated. Two clones of each cell line were used. Mean (*n* = 3) is plotted. Error bars show standard deviation. *, *P* < 0.05; **, *P* < 0.005; ***, *P* < 0.0005. (D) Western blot assays in which the indicated antibodies were used to assay levels of endogenous and exogenous ENZ and visualize the methyl landscape of indicated cell lines. * marks a nonspecific band recognized by the anti-ENZ antibody. HSP70 is a load control. (E) Virulence assay. Mice were infected with the WT or ENZ KO strain. Time of death postinfection is plotted.

We began by establishing whether *Tb*PRMT1 is essential for T. brucei survival in the mammalian host. As an RNAi-based knockdown had no effect on BF growth *in vitro* (data not shown), we generated a *Tb*PRMT1 knockout (KO) cell line in BF T. brucei to unequivocally determine whether the enzyme plays a role in virulence. Two alleles of the *Tb*PRMT1 ENZ subunit were replaced with blasticidin and puromycin resistance cassettes, whose incorporation into the genome was confirmed by PCR on genomic DNA using both *Tb*PRMT1 untranslated region (UTR)-based and open reading frame (ORF)-based primers (Fig. 1A). Growth of wild-type (WT) and KO cell lines was then measured over the course of 11 days, and a mild but reproducible growth phenotype was observed (Fig. 1B). To ascertain whether this phenotype could be attributed to the loss of *Tb*PRMT1 or whether it should be ascribed to cell-line-specific differences, we complemented the KO with the ENZ subunit of *Tb*PRMT1 exogenously expressed under doxycycline control. We expressed the exogenous ENZ for 2 weeks and then monitored growth over a 3-day period. We calculated doubling times of WT, ENZ KO, and ENZ KO+AB (add-back) cell lines and determined that the mild growth phenotype could indeed be attributed to the loss of *Tb*PRMT1 (Fig. 1C). Next we confirmed that the exogenous ENZ also restores the arginine methyl landscape of BF trypanosomes (Fig. 1D). We probed WT, ENZ KO, and ENZ KO+AB cell lysates with anti-ENZ, anti-ADMA, and anti-MMA antibodies. We saw that loss of ENZ leads to a steep increase in proteins bearing the MMA mark, a phenomenon previously observed in both mammals and T. brucei (11, 21). We also observed a modest decrease in proteins recognized by the anti-ADMA antibody. It is important to note that the anti-ADMA antibody recognizes only a small number of all proteins bearing the ADMA mark in the cell. In previous studies we found that while the observed decrease of proteins recognized by this antibody is modest, the increase of proteins bearing the MMA mark is quite striking (11). Upon exogenous expression of ENZ, the ADMA profile fully corresponded to the WT landscape. The MMA profile mostly corresponded to the WT landscape. Therefore, we conclude that the changes in methyl landscape in the ENZ KO cell line can be attributed to the loss of *Tb*PRMT1. Having established the ENZ KO strain, we infected mice with either WT or ENZ KO T. brucei to determine the impact of *Tb*PRMT1 in a living system. We found that ENZ KO leads to a significantly prolonged life expectancy of T. brucei-infected mice compared to WT (Fig. 1E, *P* <0.05). Therefore, *Tb*PRMT1 contributes to T. brucei virulence through an unknown mechanism, which may be connected to the lower growth rate of ENZ KO cells *in vitro*.

### *Tb*PRMT1 knockout leads to changes in metabolic enzyme expression and changes mRNA association of a small subset of proteins.

**FIG 2:**
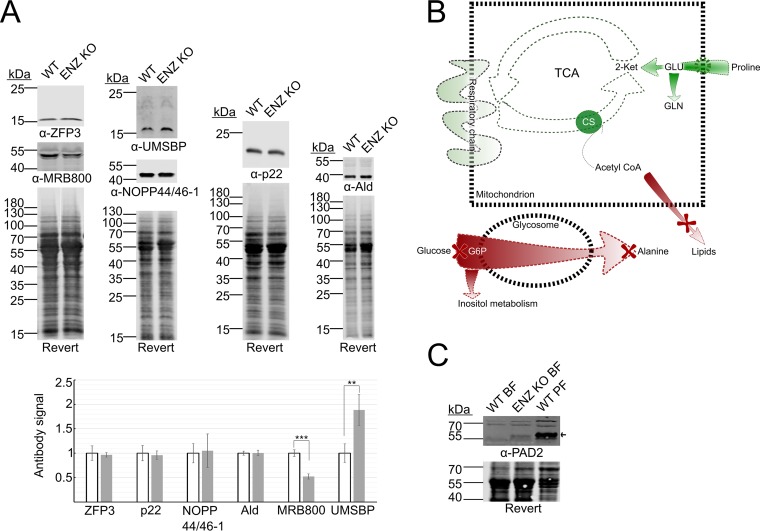
*Tb*PRMT1 knockout leads to metabolic reprogramming. (A) Western blot confirmation of changes in protein levels detected by quantitative mass spectrometry. (Top) Representative Western blot of indicated proteins. Load control, whole-protein Revert staining. (Bottom) Quantification of protein levels. An equation of trendline describing expected antibody signal per Revert signal in WT cells was obtained (Fig. S1). Expected antibody signal for each experimental sample was calculated using Revert signal. Shown is the mean from two biological and two technical replicates of actual/expected signal, with error bars denoting standard deviation. *P* values were calculated using a two-tailed *t* test. **, *P* < 0.005; ***, *P* < 0.0005. (B) Schematic representation of metabolic changes in ENZ KO cells. Green, pathways upregulated in ENZ KO. Red, pathways downregulated in ENZ KO. Red crosses indicate positions of major blocks in the pathways. Dashed green circle indicates upregulated membrane transporter. CS, citrate synthase; 2-Ket, α-ketoglutarate; GLU, glutamic acid; GLN, glutamine. (C) Western blot of bloodstream-form WT (WT BF), ENZ KO BF, and procyclic-form strain 29-13 (WT PF) cells using an antibody that recognizes PAD2. Arrow indicates PAD2 signal. Load control, whole-protein Revert staining.

RNA binding proteins are a major protein group targeted by PRMTs across the evolutionary spectrum (7 – 9, 22). Although arginine methylation of RNA binding proteins has mostly been shown to alter protein-protein interactions, examples of altered affinity for RNA upon modulation of arginine methylation state have been reported (9, 22 – 24). Since RNA binding proteins constitute the major means of regulating the T. brucei proteome, we asked whether the lack of *Tb*PRMT1 alters the association of certain proteins with mRNA. To this end, we isolated the mRNA-bound proteome from WT and ENZ KO cell lines and subjected the eluates to quantitative mass spectrometry. We anticipated that any observed changes in the mRNA-bound proteome could be attributed to either altered mRNA association or altered protein abundance in the cell. In order to discriminate between these two possibilities, we first established steady-state abundances of proteins in WT and ENZ KO cell lines by quantitative mass spectrometry analysis. We identified 4,856 proteins out of the 9,598 currently annotated in the TriTryp database as protein coding. We saw that 385 proteins decreased in abundance (>1.5-fold, *P* < 0.05) and 167 proteins increased their abundance (>1.5-fold, *P* < 0.05) in the KO compared to WT (see Table S1 in the supplemental material). We confirmed these changes by Western blotting for several proteins that did not change in abundance; MRB800, which decreased in abundance; and UMSBP1, which increased in abundance (Fig. 2A and Fig. S1). Further analysis of the data set revealed alterations in the levels of proteins involved in various aspects of T. brucei biology such as cell division, DNA repair and replication, RNA synthesis and processing, translation, the ubiquitin system, and vesicular transport. We also noted numerous remarkable alterations in the abundances of proteins involved in energy metabolism, some of which suggested possible pathway coregulation and life cycle-specific regulation. For example, ENZ KO leads to upregulation of several enzymes in the proline degradation pathway (Fig. 2B and Table 1). Interestingly, the mRNAs encoding all of the enzymes needed to obtain energy from the oxidation of proline to succinate are bound by a common RBP, DRBD3 (25). We identified significant overlap between DRBD3-bound transcripts and proteins upregulated in ENZ KO (*P* = 0.009), suggesting that this RBP may mediate a subset of the changes observed in Table 1. The proline degradation pathway is typically utilized in PF T. brucei cells that lack access to glucose (5), leading us to examine other metabolic pathways whose utilization is altered during the T. brucei life cycle. We observed an upregulation of certain subunits of respiratory chain complexes and farnesyl pyrophosphate synthase, which is involved in synthesis of CoQ (Table 1). The most prominent increase in abundance was noted in the citric acid cycle, namely, in the level of citrate synthase, which increased 10-fold. We also noted a marked decrease in enzymes participating in the major pathway of energy production in BF T. brucei: glycolysis. Specifically, hexokinase, the enzyme responsible for glucose commitment to glycolysis, decreased in abundance to about 50% of WT levels. Similar decreases were noted in the levels of glucose-6-phosphate isomerase, an enzyme working directly downstream of hexokinase, and in alanine aminotransferase. Alanine aminotransferase is responsible for conversion of pyruvate to alanine, a pathway that is extensively utilized in BF trypanosomes (26). We also noticed a decrease in several enzymes with putative roles in inositol metabolism (Table 1, “other metabolism-related genes”). Three out of four T. brucei fatty acyl CoA synthetases and glycerol-3-phosphate acyltransferase decreased in abundance, indicating a decrease in the rate of *de novo* lipid synthesis that utilizes free fatty acids and glycerol. Possibly related to these changes could be the roughly 50% decrease in aquaglyceroporin 2 (AQP2) channel abundance. AQP2 is a glycerol transporter residing in the T. brucei cell membrane that has been linked to drug resistance in African trypanosomes (27).

**TABLE 1:** Metabolism-related proteins that were identified by mass spectrometry as having altered abundance in ENZ KO cell line

Gene ID	Product name	KO/WT	*P* value
Proline metabolism			
*Tb*927.10.13120	*Tb*MCP14 mitochondrial proline transporter	2.13	4.25E−02
*Tb*927.7.210	Proline dehydrogenase	1.95	1.01E−05
*Tb*927.9.5900	Glutamate dehydrogenase	1.77	3.93E−04
*Tb*927.11.9980	Oxoglutarate dehydrogenase E1 component	1.58	1.96E−03
Glucose metabolism			
*Tb*927.10.2020	Hexokinase (*Tb*HK2)^*a*^	0.43	7.63E−03
*Tb*927.1.3830	Glucose-6-phosphate isomerase	0.58	9.28E−05
*Tb*927.1.3950	Alanine aminotransferase	0.62	4.38E−03
TCA cycle and respiration			
*Tb*927.11.9980	Oxoglutarate dehydrogenase E1 component	1.58	1.96E−03
*Tb*927.10.13430	Citrate synthase	10.90	1.18E−02
*Tb*927.10.14000	Aconitase	1.87	1.39E−03
*Tb*927.9.5960	Succinate DH-complex II	1.85	1.55E−02
*Tb*927.8.3380	Succinate DH-complex II	1.88	2.63E−03
*Tb*927.2.4700	Succinate DH-complex II	1.52	1.67E−02
*Tb*927.10.3040	Succinate DH-complex II	1.94	2.09E−02
*Tb*927.10.12540	Complex I subunit	2.76	1.78E−02
*Tb*927.10.13620	Complex I subunit	0.39	6.51E−03
*Tb*927.7.6350	Complex I subunit	5.10	7.17E−03
*Tb*927.11.15820	Complex I subunit	1.95	1.26E−02
*Tb*927.11.6980	Complex I subunit	0.40	1.91E−04
*Tb*927.1.4100	Complex II subunit	1.69	6.97E−03
*Tb*927.9.14200	Complex II subunit	2.86	3.61E−02
*Tb*927.2.3610	Complex V subunit^*a*^	1.98	2.38E−02
Other metabolism-related genes			
*Tb*927.7.3360	Farnesyl pyrophosphate synthase	4.71	6.95E−03
*Tb*11.v5.0178	Glutamine synthetase	4.59	7.32E−04
*Tb*927.8.6170	Transketolase^*a*^	2.67	2.71E−04
*Tb*927.3.4850	Mitochondrial enoyl-CoA hydratase	2.37	1.35E−02
*Tb*927.10.3100	Glycerol-3-phosphate acyltransferase^*a*^	1.70E−06	6.77E−03
*Tb*927.9.4190	Fatty acyl CoA synthetase 1	0.60	7.82E−06
*Tb*927.9.4200	Fatty acyl CoA synthetase 2	0.63	2.70E−06
*Tb*927.9.4210	Fatty acyl CoA synthetase 3	0.39	4.38E−04
*Tb*927.10.14170	Aquaglyceroporin 2	0.46	1.59E−05
*Tb*927.9.6350	Inositol monophosphatase^*a*^	5.72E−04	3.58E−02
*Tb*927.3.4570	*N*-Acetylglucosamyl transferase component GPI1	0.01	3.94E−02
*Tb*927.10.4780	GPI inositol deacylase	0.22	4.07E−02
*Tb*927.3.2610	GPI inositol deacylase 2	0.32	1.87E−04
*Tb*927.8.6390	Lysophospholipase	0.62	2.52E−02
*Tb*927.10.6440	Phosphomannomutase	0.67	3.67E−02

a Arginine methylation identified in proteome-wide screen (7, 8; unpublished data).

10.1128/mBio.02430-18.2 FIG S1 Determination of antibody linear range. Increasing amounts of BF T. brucei cell lysate were probed with indicated antibodies. Signals of both antibody and Revert (LC) were normalized to the signal in the first lane and plotted. Trendline was plotted, and equation of the trendline was used to determine values in Fig. 2A. Download FIG S1, PDF file, 0.3 MB. This is a work of the U.S. Government and is not subject to copyright protection in the United States. Foreign copyrights may apply.

10.1128/mBio.02430-18.3 TABLE S1 Whole-cell quantitative mass spectrometry analysis of BF wild-type and ENZ KO cells. All identified proteins are shown in the “Identified proteins” tab. Proteins with change of >1.5× and *P* < 0.05 are considered significantly changed. “Increased in KO” and “Decreased in KO” tabs contain the two subsets of changed proteins. Download Table S1, XLSX file, 1.3 MB. This is a work of the U.S. Government and is not subject to copyright protection in the United States. Foreign copyrights may apply.

Overall, the observed changes in metabolism resembled those typically observed during the transition from BF to PF. However, when we compared the entire data set to proteins previously shown to be up-/downregulated in PF or BF, we did not see a significant overlap with those that change upon ENZ KO (28). We did, however, observe an increase in PAD2, a protein involved in conveying the differentiation signal in the BF-to-PF life cycle transition (29). PAD2 is absent from the long slender BF stage, increases in abundance in the stumpy stage, and remains on the surface once the cells progress to the PF stage. We wanted to verify the increase in PAD2 in BF upon ENZ KO through Western blotting using anti-PAD2 antibodies (29). We were unable to quantitate the increase due to the absence of PAD2 signal in our WT BF cells. However, we do clearly observe a detectable amount of PAD2 in ENZ KO cells, validating the increase in this key differentiation molecule (Fig. 2C). Overall, we conclude that the loss of ENZ leads to a dysregulation of T. brucei metabolism. This effect may be achieved through regulatory mechanisms that play a role in life cycle progression or alternatively may be a compensatory effect as the cells cope with the stress of ENZ loss.

**FIG 3:**
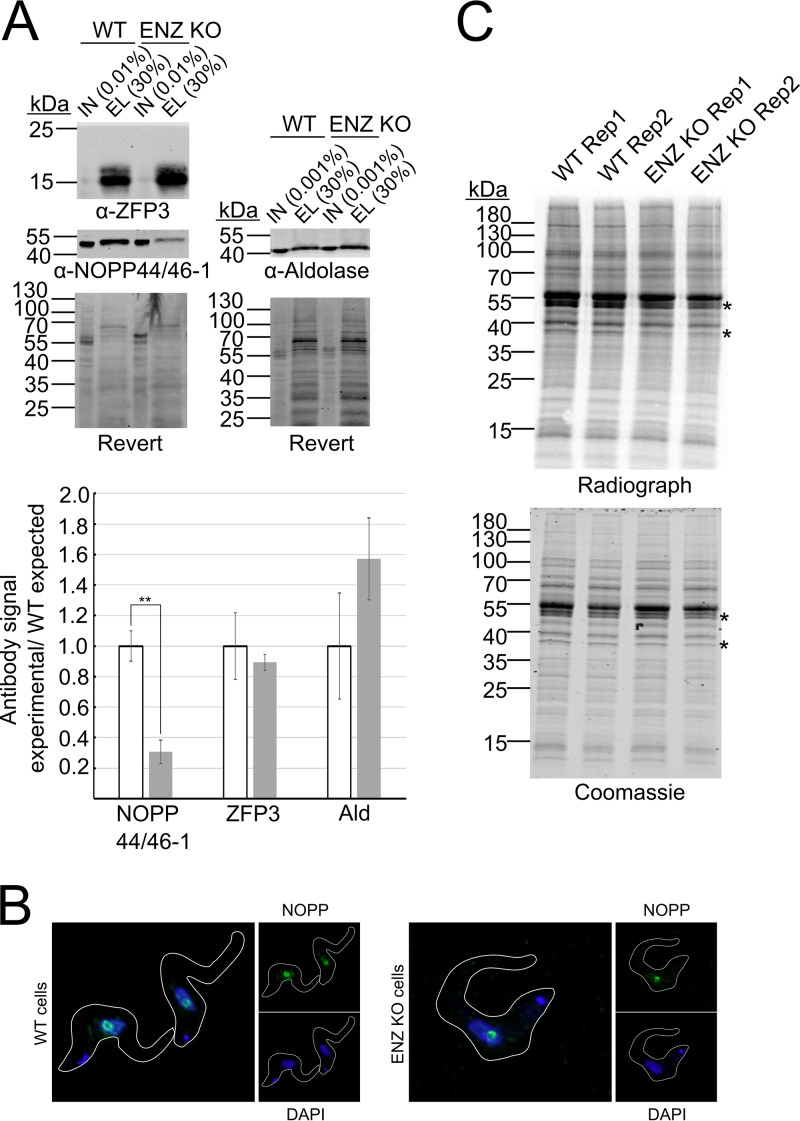
Confirmation of mRNA-bound proteome changes. (A) Western blot confirmation of mRNA-bound protein levels. (A) (Top) Representative Western blot of indicated proteins. IN, input; EL, elution. Indicated is % of total sample. Load control, whole-protein Revert staining. (Bottom) Quantification of protein levels. Antibody signals were normalized to Revert and to the mean signal of the WT sample. Mean from biological triplicates is plotted with error bars denoting standard deviation. *P* values calculated using two-tailed *t* test. **, *P* < 0.005. (B) Immunolocalization of NOPP44/46-1 by immunofluorescence with a NOPP44/46-1-specific antibody. Large panel shows merged DAPI and NOPP44/46-1 signal. (C) [^35^S]methionine labeling of nascent proteins. Labeled cells were lysed, and proteins were separated by SDS-PAGE. Coomassie, protein load; Radiograph, nascent protein signal. * indicates proteins that changed expression.

Having established steady-state levels of proteins in our WT and ENZ KO cell lines, we proceeded to quantify changes in the mRNA-bound proteome. WT and KO cells were UV cross-linked, and mRNA was affinity purified using oligo(dT)-coated magnetic beads (30). The mRNA with bound proteins was eluted off the beads, and proteins were quantified by mass spectrometry (Table S2). We identified 234 proteins, including 129 out of 155 proteins that were previously identified as mRNA binding in BF T. brucei by Lueong et al. (30). We observed significant changes in the abundance of 16 proteins (>1.5-fold, *P* < 0.05) in ENZ KO compared to WT parasites (Table 2). Of these, 14 proteins were also identified in our whole-cell proteome analysis (Table S1). Only the levels of RBP35 (*Tb*927.9.12360) and Nopp44/46-2 (*Tb*927.8.750) significantly changed on the whole-cell level, although eight proteins showed a trend that suggested a possible change but did not pass the significance criteria (Table 2). To verify the mRNA-bound proteome data set, we repeated the mRNA purification experiment in biological triplicate and detected selected proteins by Western blotting (Fig. 3A). Based on available antibodies, we chose to assay levels of ZFP3 (*Tb*927.3.710, unchanged), NOPP44/46-1 (*Tb*927.8.760, decreased), and aldolase (*Tb*927.10.5620, increased). The aldolase Western blot did not reveal a significantly increased association with mRNA in the ENZ KO cell line, although the quantification showed a trend consistent with a slight increase. This result was not surprising considering that, unlike ZFP3 and NOPP44/46-1 proteins, which are both involved in nucleic acid metabolism, aldolase is an extremely abundant metabolic enzyme. Thus, the increased mRNA association of aldolase in our experiment may be due to contamination of *Tb*PRMT1 KO samples. In contrast, Western blot analysis confirmed our mass spectrometry results for ZFP3 and NOPP44/46-1, demonstrating unchanged levels for the former and a decrease of the latter to less than 30% of WT levels. Interestingly, we previously showed that NOPP44/46-1 is heavily decorated by ADMA, consistent with an important role for *Tb*PRMT1 in its activity (7).

**TABLE 2:** Proteins changing their mRNA association in ENZ KO cells

Protein ID	Bound to mRNA		Whole-cell proteome		Lueong et al. (30) FDR	Product description^*a*^
	KO/WT ratio	*P* value	KO/WT ratio	*P* value		
*Tb*927.10.5620	6.0	6.E−04	1.00	0.84	0.18	Fructose-bisphosphate aldolase, glycosomal
*Tb*927.10.4430	2.0	3.E−02	NA	NA	3.3E−03	PUF1, posttranscriptional repressor
*Tb*927.9.10400	1.8	2.E−02	0.92	0.11	8.1E−03	Nucleolar protein of unknown function
*Tb*927.10.10280	1.7	1.E−02	1.22	1.E−02	4.4E−02	*Tb*BBP268
*Tb*927.9.4080	0.6	5.E−03	1.13	0.16	2.5E−03	Stumpy formation signaling pathway protein HYP2, posttranscriptional activator
*Tb*927.6.5010	0.6	8.E−03	0.50	0.15	5.3E−03	Putative nuclear protein, posttranscriptional repressor
*Tb*927.9.12360	0.6	3.E−03	0.58	6.E−03	6.3E−03	RBP35, posttranscriptional repressor
*Tb*927.11.10810	0.5	2.E−03	NA	NA	8.7E−03	PUF11
*Tb*927.9.15290	0.6	3.E−03	0.25	0.42	1.0E−02	CHAT domain-containing protein, putative
*Tb*927.8.710	0.7	1.E−02	0.01	0.14	2.3E−02	DRBD17
*Tb*927.10.7310	0.1	2.E−04	0.39	0.59	2.7E−02	Terminal uridylyltransferase 3
*Tb*927.8.750	0.6	1.E−02	1.90	6.E−09	3.3E−02	NOPP44/46-2
*Tb*927.8.760	0.4	8.E−03	1.12	0.41	5.0E−02	NOPP44/46-1
*Tb*927.11.4380	0.4	4.E−02	0.09	0.15	NA	ATP-dependent RNA helicase, putative
*Tb*927.8.4500	0.5	1.E−03	0.36	0.15	3.2E−04	eIF4G5, posttranscriptional activator
*Tb*927.11.14590	0.4	4.E−04	0.37	0.16	1.2E−02	eIF4G5-interacting protein

a Posttranslational activator/repressor function determined by RNA tethering screen in Erben et al. (53).

10.1128/mBio.02430-18.4 TABLE S2 Quantification of overall association of proteins with mRNA in WT and ENZ KO BF T. brucei cells. Proteins with change of >1.5× and *P* < 0.05 are considered significantly changed. Download Table S2, XLSX file, 0.1 MB. This is a work of the U.S. Government and is not subject to copyright protection in the United States. Foreign copyrights may apply.

Having verified the data set, we more closely analyzed the proteins exhibiting altered mRNA association in ENZ-depleted cells. We first asked whether these proteins have commonalities in their subcellular localization by examining data from the TrypTag project. Interestingly, out of the 8 proteins tagged to date, 7 localize at least partially to cytoplasmic punctae in PF T. brucei (*Tb*927.10.4430, *Tb*927.9.4080, *Tb*927.9.12360, *Tb*927.11.10810, *Tb*927.8.710, *Tb*927.8.4500, and *Tb*927.11.14590). We were also intrigued by the change in mRNA association of eIF4G5, its interacting protein G5-IP, and two members of the NOPP44/46 family, given the reported translation-related functions of these proteins. eIF4G5 forms a complex with eIF4E6 and G5-IP, a protein with similarity to capping enzymes. This complex has affinity for capped mRNAs, but its depletion does not significantly decrease the overall translation rate (31). NOPP44/46 is a trypanosome-specific protein family that bears extensive, life cycle-regulated tyrosine phosphorylation and has been implicated in ribosomal 60S subunit maturation (32). Since NOPP44/46-1 is known to shuttle between nucleus and cytoplasm, we first wanted to determine whether the decrease in NOPP44/46-1 mRNA binding could be due to mislocalization (33). We immunolocalized the protein but did not observe any change between the WT and ENZ KO cell lines (Fig. 3B). Given the potential roles of the above-mentioned proteins in translation, we next asked whether ENZ KO leads to an altered translation rate of all or a subset of proteins. We pulsed the cells with [^35^S]methionine and separated the nascent proteins by SDS-PAGE. We detected a reproducible decrease in translation of an ∼50-kDa and an ∼38-kDa protein but did not see any overall translation defect (Fig. 3C). It is important to note that this assay lacks the resolution to visualize low-abundance proteins; therefore, it is quite possible that the number of proteins whose translation rate is affected exceeds the two easily visualized proteins. We conclude that the loss of *Tb*PRMT1 alters the mRNA binding capacity of a small number of cytoplasmic proteins, which in turn may lead to specific impacts on mRNA translation.

### *Tb*PRMT1 is necessary for an efficient T. brucei starvation stress response.

**FIG 4:**
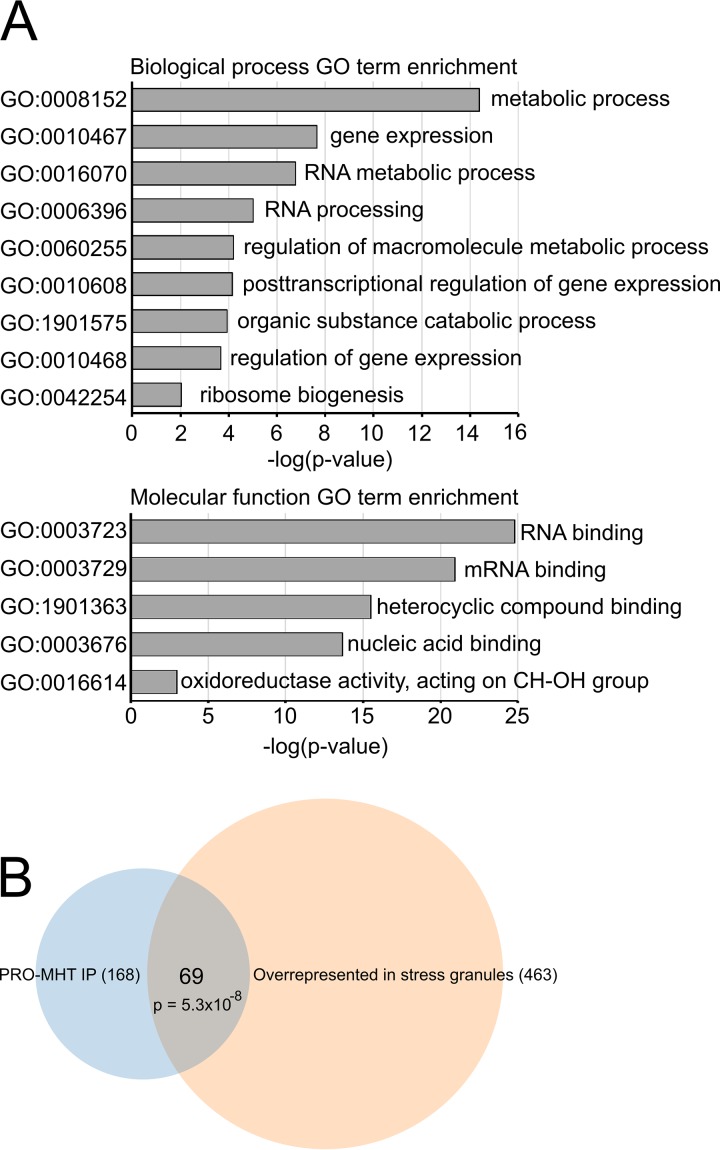
Proteins identified in PRO-MHT purification. (A) GO term enrichment was acquired from TriTrypdb.org. *P* values are Bonferroni corrected. (B) Venn diagram of proteins present in PRO purification and proteins overrepresented in stress granules (34). *P* value calculated using R p-hyper function.

**FIG 5:**
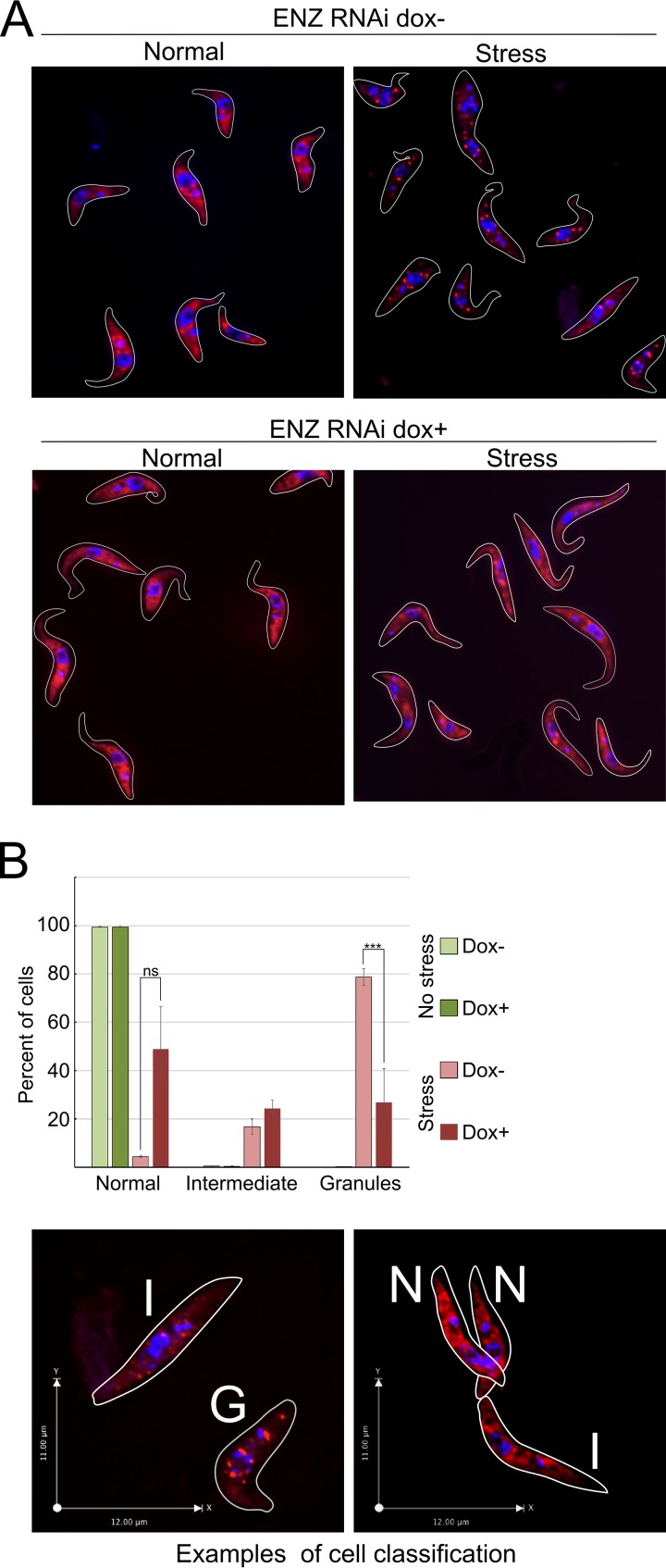
*Tb*PRMT1 is necessary for mRNA migration to starvation stress granules. (A) mRNA was visualized by oligo(dT) FISH in the PF ENZ RNAi cell line. Uninduced cells (dox-) or RNAi-induced cells (dox+) were either cultured in medium (Normal) or nutritionally starved (Stress). mRNA was visualized by FISH (red), and DNA was stained with DAPI (blue). (B) Cells were divided into normal (N), intermediate (I), or granule (G) categories (for examples, see bottom panel) and counted. Means for two biological replicates of 300 to 500 cells each were plotted, and *P* values were calculated using the chi-squared test. ***, *P* < 0.0005. Error bars show the range of values from each experiment.

To begin to distinguish secondary effects of *Tb*PRMT1 depletion from the primary affected pathways, we need to determine possible *Tb*PRMT1 substrates. Since our antibodies are not specific enough to immunoprecipitate either ENZ or PRO subunit, we attempted to purify exogenously expressed PRO-Myc-His-TAP (PRO-MHT) from BF T. brucei to identify associated proteins that may be substrates. We were unable to achieve sufficient expression for proteomic analysis in BF and so switched to PF. We successfully expressed PRO-MHT and purified associated proteins (see Table S3 in the supplemental material). Complexes were eluted by TEV protease cleavage to minimize contaminants, and cells in which PRO-MHT expression was not induced were used as a negative control. We refined our data set by eliminating all proteins that were identified by <2.5× peptides over the negative control, using the replicate with lower peptide count for that specific protein. Applying this criterion, we identified enriched biological process GO terms for our data set. We observed a significant enrichment for proteins involved in metabolic processes and various levels of gene expression, findings that supported our previous data indicating *Tb*PRMT1 involvement in regulation of T. brucei metabolism (Fig. 4A). Unsurprisingly, when we looked at molecular function GO terms, the overwhelming majority of identified proteins fell into the RNA binding protein category (Fig. 4A). A single protein was identified in common between the proteins that changed their mRNA association and proteins that copurify with *Tb*PRMT1: a nucleolar protein with unknown function (*Tb*927.9.10400). Although not identified as methylated on arginine residues in our proteome-wide screen (7, 8; also unpublished data), the *Tb*927.9.10400 sequence contains multiple RGG motif repeats, which are typically targeted by arginine methylation, making *Tb*927.9.10400 a promising target for future studies. One finding that caught our eye was the significant overlap of proteins that copurify with *Tb*PRMT1 and proteins that are enriched in T. brucei starvation granules (34) (Fig. 4B) (*P* = 5.3 × 10^−8^) (Table 3). This includes SCD6 (*Tb*927.11.550), a stress-related protein that was previously reported to copurify with *Tb*PRMT1 (35). Association of *Tb*PRMT1 with starvation stress-related granules was particularly striking since the PRO subunit of *Tb*PRMT1 itself was enriched in a recent proteomic analysis of stress granules (34). To investigate a possible role of *Tb*PRMT1 in stress granule formation, we utilized a PF ENZ RNAi cell line that efficiently ablates PRO and ENZ protein levels when induced by doxycycline (11). Either uninduced or RNAi-induced cells were starved by a two-hour incubation in PBS, and mRNA was subsequently visualized by oligo(dT) fluorescent *in situ* hybridization (FISH) (Fig. 5A). We classified the cells as either stressed (clearly containing RNA granules), intermediate (some granulation might be present), or normal (mRNA distributed evenly throughout cytoplasm) (Fig. 5B, lower panel). We observed a statistically significant decrease in cells that were able to form stress granules upon *Tb*PRMT1 depletion (Fig. 5B, upper panel). Together, these data demonstrate a role for *Tb*PRMT1 in starvation stress granule formation in PF T. brucei and suggest potential substrates that may contribute to this effect.

**TABLE 3:** Stress-granule enriched RNA binding proteins present in PRO-MHT purification^*a*^

Protein ID	No. of peptides identified			Product name	R methylation
	Rep1	Rep2	Neg ctrl		
*Tb*927.10.3560	501	622		PRO	
*Tb*927.1.4690	216	274		ENZ	
*Tb*927.9.12510	20	35	5	ATP-dependent DEAD/H RNA helicase	ADMA/DMA/MMA
*Tb*927.11.550	20	31	8	Hypothetical protein SCD6.10	
*Tb*927.10.2370	19	15		Lupus La protein homolog	
*Tb*927.4.2040	15	15		DNA/RNA-binding protein Alba 3	DMA/MMA
*Tb*927.9.13990	14	17		RNA-binding protein, putative	
*Tb*927.11.14220	12	17	3	Hypothetical protein, conserved	
*Tb*927.10.6060	11	9		Universal minicircle sequence binding protein 2	
*Tb*927.11.2250	6	24		HYP11	ADMA
*Tb*927.10.6050	6	16		Clathrin heavy chain	
*Tb*927.10.11760	5	8		Pumilio/PUF RNA binding protein 6	ADMA
*Tb*927.7.4900	4	7		5′–3′ exoribonuclease A	SDMA/MMA
*Tb*927.6.640	4	6		ApaH-like phosphatase ALPH1	
*Tb*927.8.990	3	8		RNA-binding protein 33	ADMA/SDMA
*Tb*927.10.14950	3	7		Zinc finger CCCH domain-containing protein 40	
*Tb*927.6.1870	3	5		Eukaryotic translation initiation factor 4E-4	
*Tb*927.4.410	2	5		CAF 40	
*Tb*927.8.1500	2	3		Hypothetical protein, conserved	

a DMA, dimethylarginine (ADMA/SDMA could not be determined); ADMA, asymmetric dimethylarginine; SDMA, symmetric dimethylarginine; MMA, monomethylarginine.

### *Tb*PRMT1 directly binds to nucleic acid.

**FIG 6:**
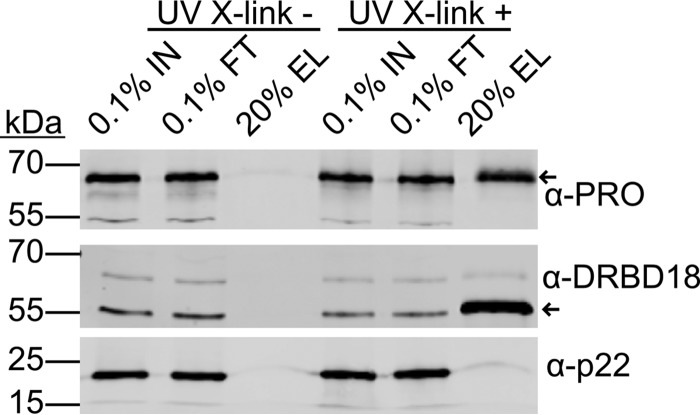
*Tb*PRMT1 binds mRNA *in vivo.* mRNA was purified from PF cells expressing PRO-MHT, which were either left untreated (UV X-link -) or UV cross-linked (UV X-link +) prior to lysis. Western blotting was used to detect the indicated proteins in input (IN), oligo(dT) bead flowthrough (FT), and oligo(dT) bead elution (EL). Percentages loaded are indicated. Positive control, DRBD18; negative control, p22. Arrows indicate migration of target proteins.

**FIG 7:**
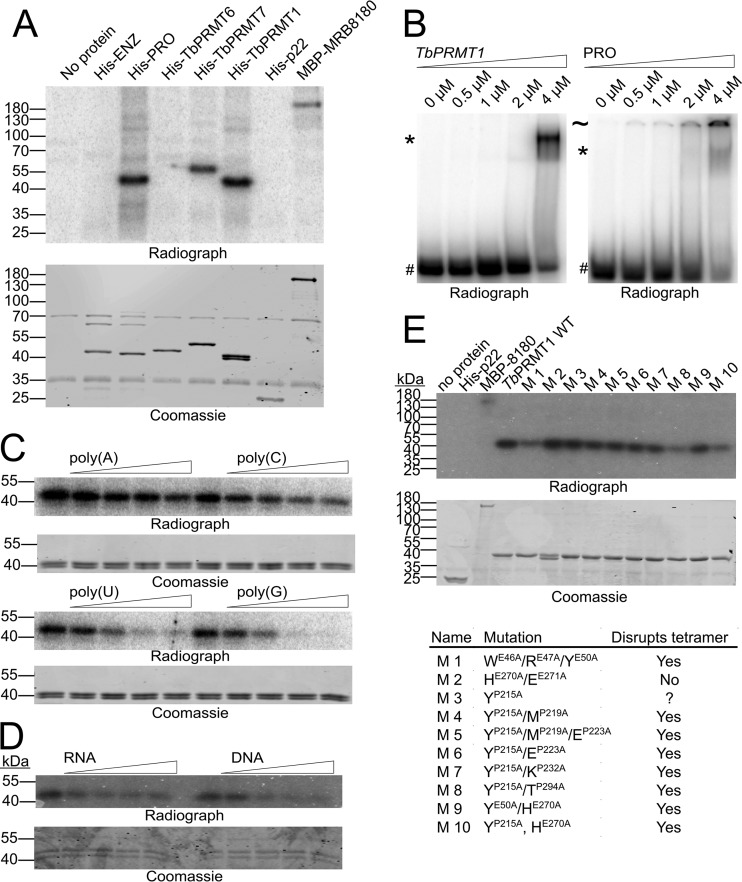
*Tb*PRMT1 binds mRNA *in vitro*. (A) ^32^P-labeled pBluescript SK− RNA was cross-linked to equimolar amounts of the indicated recombinant proteins. PRMT molarity was calculated per dimer. Reaction mixtures were treated with RNase A and resolved by SDS-PAGE. The dried, Coomassie blue-stained gel was exposed to a phosphorimager screen (Radiograph). Positive control, MBP-MRB8180; negative control, His-p22. (B) ^32^P-labeled pBluescript SK− RNA was incubated with increasing amounts of recombinant *Tb*PRMT1 or PRO. Reaction mixtures were resolved by native PAGE, and the dried gel was visualized by phosphorimager screen exposure. #, free RNA; *, PRMT/RNA complex; ∼, possible PRMT/RNA aggregate. (C) UV cross-linking assay performed as in panel A in the presence of unlabeled oligo(N) competitor. (D) UV cross-linking assay performed as in panel A in the presence of unlabeled RNA/DNA competitor. (E) UV cross-linking assay performed as in panel A utilizing a battery of *Tb*PRMT1 mutants. Table at bottom of panel E describes the mutations. P or E in the superscript designates whether the mutation was in the PRO or ENZ subunit, respectively.

The effect of *Tb*PRMT1 on stress granule formation led us to investigate the possibility that *Tb*PRMT1 might itself be able to associate with RNA. This notion was supported by a recently reported mRNA-bound proteome study in which more PRO peptides were purified with the mRNA than in the negative control, although the significance criterion was not met (30). To investigate whether *Tb*PRMT1 has RNA binding properties, we began by analyzing the association of *Tb*PRMT1 with mRNA *in vivo*. We attempted to use our antipeptide *Tb*PRMT1 antibodies; however, the results were inconclusive due to the interference of nonspecific bands (data not shown). Therefore, we utilized our PRO-MHT cell line to purify the mRNA-bound proteins. Due to the presence of the protein A moiety in the TAP tag, the secondary anti-rabbit IgG antibody strongly recognized the tagged protein (Fig. 6). We saw a clear association of PRO-MHT with mRNA as well as the presence of our positive control, DRBD18. Our negative control, p22, was not present in the final eluate. Therefore, we conclude that PRO associates with mRNA *in vivo*. To confirm that PRO directly associates with RNA, we turned to *in vitro* methods. Since our attempts to identify *in vivo Tb*PRMT1 mRNA targets were unsuccessful, we utilized a 102-nt ^32^P-labeled pBluescript SK− plasmid RNA. We generated recombinant *Tb*PRMT1 heterotetramer by ENZ and His-PRO coexpression in Escherichia coli followed by nickel affinity purification of the complex as described previously (12). Individual *Tb*PRMT1 subunits were expressed as His-ENZ or His-PRO separately and purified. We incubated the RNA with *Tb*PRMT1, individual *Tb*PRMT1 subunits, and other T. brucei PRMTs; UV cross-linked the samples; separated them by SDS-PAGE; and visualized RNA-bound complexes (Fig. 7A). We observed no RNA binding when only the ENZ *Tb*PRMT1 subunit was present, although this result should be viewed with caution, since the ENZ subunit is difficult to purify by itself and is prone to aggregation. Importantly, we saw clear RNA binding of the PRO subunit both incubated alone with RNA and within the context of the entire *Tb*PRMT1 heterotetramer. We also observed binding of *Tb*PRMT7, a type III PRMT that was enriched in the published T. brucei mRNA-bound proteome to a similar degree as *Tb*PRMT1 (30). *Tb*PRMT6 was not found cross-linked to RNA, suggesting that *in vitro* RNA binding is not a default feature of all PRMTs. Next, we wanted to utilize another, more native method to confirm that *Tb*PRMT1 binds RNA *in vitro*. We incubated increasing concentrations of *Tb*PRMT1 or its PRO subunit with labeled RNA and resolved the reaction mixtures by native PAGE, resulting in an electrophoretic mobility shift (EMSA) of protein-bound RNA. We saw a distinct shift of RNA migration at the highest protein concentrations (2 and 4 μM) (Fig. 7B). This suggests a relatively low affinity of *Tb*PRMT1 for pBluescript RNA but confirms *Tb*PRMT1 RNA binding ability. Some proteins exhibit affinity for specific polynucleotide sequences, and the *in vivo* mRNA binding activity of *Tb*PRMT1 suggested it could exhibit high affinity for poly(A). To investigate whether *Tb*PRMT1 has a preference for specific polynucleotides, we performed the *in vitro* RNA cross-linking assay using a 47-nt transcript of pBluescript sequence in the presence of increasing amounts of unlabeled poly(A), poly(C), poly(U), and poly(G) to determine their abilities to compete with the labeled probe for binding to *Tb*PRMT1 (Fig. 7C). In these assays, PRO exhibited a preference for poly(U) and poly(G) but little binding to poly(A) or poly(C). Considering the low affinity of *Tb*PRMT1 for RNA, we wanted to determine whether *Tb*PRMT1 binds RNA specifically or whether the binding ability extends to all nucleic acids. We incubated *Tb*PRMT1 with labeled RNA in the presence of unlabeled RNA or DNA competitor. We saw that the two competitors performed equally; therefore, we conclude that *Tb*PRMT1 has nucleic acid binding ability (Fig. 7D). In another study (H. Hashimoto, L. Kafková, K. Jordan, L. K. Read, and E. W. Debler, submitted for publication), we determined that *Tb*PRMT1 tetramerization is necessary for *Tb*PRMT1 methylation activity. We thus wanted to test whether the tetramerization also influences *Tb*PRMT1 RNA binding properties. We performed the RNA cross-linking assay on a battery of mutants that were unable to tetramerize (Fig. 7E). We observed that while tetramerization is not required for RNA binding, specific mutations in either the ENZ or PRO subunits of *Tb*PRMT1 decrease the RNA binding potential of the protein complex. The finding that specific mutations are able to decrease *Tb*PRMT1 RNA affinity strongly suggests that *Tb*PRMT1 RNA binding is a specific and biologically relevant property of the enzyme, and it opens a window to further studies that will determine the residues directly involved in *Tb*PRMT1 RNA binding.

## DISCUSSION

Arginine methylation is a ubiquitous posttranslational modification that influences a broad spectrum of eukaryotic cellular processes (36 – 41). The enzymes catalyzing this modification, PRMTs, are astonishingly well conserved throughout the eukaryotic kingdom. The enzymatic subunit of *Tb*PRMT1, for example, shares 53% amino acid sequence identity with human PRMT1, a homology quite unusual between organisms with such evolutionary distance between them. In this study, we provide biochemical and cell biological characterization of *Tb*PRMT1. We show that in BF T. brucei, *Tb*PRMT1 promotes virulence in the animal model, influences energy metabolism, and changes mRNA association of a small subset of proteins. In insect-stage parasites, loss of *Tb*PRMT1 significantly hinders the parasite’s ability to form mRNA-containing cytoplasmic granules as a response to starvation stress. Furthermore, we show that *Tb*PRMT1 is itself able to bind nucleic acids both *in vitro* and *in vivo*.

The virulence decrease of ENZ KO trypanosomes is interesting in the context of a phenotype observed in Leishmania major, a parasite closely related to *Trypanosoma* species. In contrast to what we observed in *Tb*PRMT1 KO parasites, deletion of the *Lmj*PRMT7 gene led to an increase in virulence, while overexpression caused decreased lesion progression in a mouse model (42). PRMTs are well known to engage in interplay in both T. brucei and human cells (11, 21). Depletion of PRMT1 in both cell types causes a massive increase in MMA, which in T. brucei PF cells is attributed to *Tb*PRMT7 activity (11). While the opposing effects of downregulating the two proteins could be a coincidence, especially considering that the data originated from different species, it is plausible that the virulence decrease we observe in the absence of *Tb*PRMT1 results from increased *Tb*PRMT7 activity.

Trypanosomes extensively modulate their energy metabolism throughout their life cycle as well as in response to available nutrients (43, 44). In ENZ KO cells, we observed a number of alterations in metabolic enzyme abundance that are reminiscent of metabolic changes observed during T. brucei life cycle progression, namely, a decrease in glycolytic enzymes and an increase in components of the citric acid cycle and the proline degradation pathway. This prompted us to assess the ability of ENZ KO cells to undergo citrate-/*cis*-aconitate-induced *in vitro* differentiation from BF to PF. We did not observe any difference in the rate at which the cells acquired a procyclin coat or lost VSG coating compared to WT parasites (data not shown). This result could suggest that the observed changes are a result of slow growth rather than a reflection of interference with life cycle progression regulation (45, 46). However, it is important to note that the citrate/*cis*-aconitate differentiation method forces the parasites to progress from BF to PF, skipping the stumpy stage, which our laboratory strain is not able to produce. Both the observed ∼50% decrease in mRNA binding of HYP2 (*Tb*927.9.4080), a protein identified in a screen aimed at factors driving stumpy formation, and the increase in PAD2 protein levels in ENZ KO cells suggest possible *Tb*PRMT1 involvement in BF to stumpy-stage progression (4). Furthermore, the observed defect in starvation granule formation in *Tb*PRMT1-depleted cells hints at a possible defect in a life cycle progression through impaired response to the nutrient-poor environment that is naturally found in fly midgut and salivary glands. It is known that the differentiation of Trypanosoma cruzi, a close relative of T. brucei, from proliferative epimastigotes to infective growth-arrested metacyclic trypomastigotes requires nutritional stress as one of the signals (47, 48). To better understand the role of *Tb*PRMT1 in the life cycle progression of T. brucei, it will be necessary to generate a pleomorphic T. brucei cell line lacking *Tb*PRMT1 and observe the ability of these cells to progress through the natural life cycle in the animal and fly hosts.

To begin understanding the direct effects of *Tb*PRMT1-mediated methylation, it is important to identify *Tb*PRMT1 substrates. Some of the observed effects are likely caused by regulation of RNA binding proteins either by direct methylation or by methylation of their binding partners. Our data show changes in the mRNA association of several RNA binding proteins. PUF1 and *Tb*927.9.10400 exhibit increased association with RNA in the absence of *Tb*PRMT1. PUF1 depletion causes only minor changes in PF and BF gene expression, although this was investigated only by the relatively insensitive microarray approach, and the role of PUF1 in other T. brucei life cycle stages was not investigated (49). PUF1 is unlikely to be a direct target of *Tb*PRMT1, since our proteome-wide screens did not identify arginine methylation of PUF1, nor was PUF1 found associated with *Tb*PRMT1 (7, 8; unpublished data). *Tb*927.9.10400 is a nucleolar protein of unknown function (50). Despite the absence of *Tb*927.9.10400 in our proteome-wide methylation screen, the protein sequence contains numerous RGG repeats that are typically targeted by arginine methylation. Furthermore, *Tb*927.9.10400 was in the top 5% of PRO-MHT-interacting proteins based on the spectral count, making it a promising candidate for future studies.

None of the proteins that decreased their mRNA association upon ENZ KO were found interacting with *Tb*PRMT1. Nevertheless, the decrease in HYP2 mRNA binding that we observed in ENZ KO cells is intriguing. HYP2 contains a DksA zinc finger, a motif involved in nutritional status and quorum sensing responses in prokaryotes, and has been identified as a part of a quorum sensing signaling cascade that plays a role in stumpy-stage formation in T. brucei (4). The HYP2 protein sequence contains a number of arginines in the RG context, making it a potential target of arginine methylation. It is also possible that the HYP2-mRNA interaction is indirectly affected by *Tb*PRMT1 activity. HYP11 (*Tb*927.11.2250) was also implicated to play a role in stumpy formation (4, 51). HYP11 bears an ADMA mark, copurifies with *Tb*PRMT1, has RNA binding properties, and is enriched in stress granules (4, 30, 34), strongly suggesting that HYP11 is a primary *Tb*PRMT1 target. While HYP11 mRNA binding is not changed in our screen, it is possible that arginine methylation of HYP11 influences HYP2 mRNA binding or changes the composition of an HYP11-containing ribonucleoprotein, ultimately altering mRNA fate. It is important to note that our mRNA binding screen is likely biased toward identification of mRNA association changes in proteins with a narrow specificity toward a small subset of mRNAs, since changes in specificity of proteins with a large pool of bound mRNAs would not result in a statistically significant change in overall mRNA binding.

Other regulators of mRNA fate that could be affected by arginine methylation include DRBD3 and SCD6. DRBD3 is essential in PF cells and acts as a stabilizing factor of a small subset of mRNAs, some of which undergo stage-specific regulation (52). Considering that levels of DRBD3 remain constant between PF and BF cells, it is possible that stage-specific regulation is achieved through PTM decoration (52). Interestingly, among DRBD3 targets are the enzymes constituting the proline degradation pathway, several of which are upregulated in ENZ KO cells. DRBD3 was annotated as a posttranscriptional activator based on a high-throughput tethering assay (53). DRBD3 was not found methylated in our screen; however, it does harbor an RGG motif that is typically targeted by PRMTs. Starvation stress alters the composition of DRBD3-associated ribonucleoprotein complexes and causes DRBD3 to localize into cytoplasmic granules (54). Among DRBD3-interacting proteins are PABP1, PABP2, and NOT1, all of which copurify with *Tb*PRMT1 and are also overrepresented in starvation stress granules (54). It is plausible that DRBD3 function is regulated through methylation of these proteins. Furthermore, DRBD3 appears as a possible mechanistic link between stage-specific metabolism regulation and starvation stress response. SCD6/RAP55 is a translational repressor with a function in stress granule formation in yeast and mammals (55 – 57). SCD6 function is modulated by HMT1/PRMT1 in yeast and mammals, and in T. brucei SCD6 strongly interacts with *Tb*PRMT1 and is methylated on three arginine residues (35, 58 – 61). Unlike SCD6 in other organisms, *Tb*SCD6 does not seem to be necessary for formation of mRNA-containing granules under starvation conditions (35). However, *Tb*SCD6 is a component of processing bodies, which are constitutive components of cytoplasmic mRNA metabolism in trypanosomes with a putative role in translational repression (62). The tight association of *Tb*SCD6 with *Tb*PRMT1 suggests that *Tb*SCD6 is likely to play a role in some of the changes we observe on the proteome level.

*Tb*PRMT1 could also be involved in directly regulating the functions of metabolic enzymes in the affected pathways. Glycolysis is the main pathway that provides energy for BF T. brucei cells (63). Upon knockout of ENZ, we see approximately a 50% decrease in hexokinase 2 (*Tb*HK2) and phosphoglucose isomerase levels. We also noted a 40% decrease in levels of alanine aminotransferase, an enzyme responsible for conversion of pyruvate to alanine, a pathway that is extensively utilized in BF trypanosomes (26). Together, these data suggest a decrease in glycolytic flux, and direct measurement of the impact of *Tb*PRMT1 knockout on the glycolytic rate is a future focus. Hexokinase catalyzes a virtually irreversible reaction that commits glucose to glycolysis. T. brucei carries two genes with hexokinase homology that are 98% identical at the amino acid level, *Tb*HK1 and *Tb*HK2. Unlike in other eukaryotes where hexokinases function as dimers, *Tb*HKs form hexameric complexes, and they were isolated from cells as a heterohexamer containing an unknown ratio of *Tb*HK1 and *Tb*HK2 (64). T. brucei further differs from mammals in that *Tb*HK is not inhibited by its product, glucose-6-phosphate (65). While *Tb*HK2 is not active on its own, it gains activity when complexed with inactive *Tb*HK1 (66). This complex then becomes amenable to inhibition by pyrophosphate, a molecule that has no effect on the *Tb*HK1 homohexamer (66). These findings suggest that the two enzymes have distinct regulatory mechanisms that differ from the mammalian hexokinase regulation system. A peptide that could originate from either *Tb*HK1 or *Tb*HK2 (SKYRFVLPTTKFDLD) was shown to bear arginine dimethylation in our proteome-wide screen; therefore, *Tb*PRMT1 is likely to contribute to this intricate and unusual pathway for regulation of glycolysis.

We show here that *Tb*PRMT1 has nucleic acid binding properties and associates with mRNA *in vivo*. Future studies will be focused on determining the functional relevance of this property. One possibility is that *Tb*PRMT1 can methylate RNA as well as arginines. *Tb*PRMT1 shares features with the human RNA methyltransferase Mettl3/Mettl14. Mettl3/14 form a heterodimer in which Mettl3 is a catalytically active subunit while Mettl14 plays a critical structural role necessary for substrate recognition (67). Once *in vivo* RNA targets of *Tb*PRMT1 are identified, they can be tested as potential substrates. Another possibility is that RNA binding plays a role in defining the appropriate topology of *Tb*PRMT1 in respect to RNA-/DNA-bound substrates, as in the case of human lysine SET1 methyltransferase (68). Indeed, nucleic acid binding may be a conserved property of PRMT1 enzymes rather than a feature specific to T. brucei. PRMT1 was identified among the mRNA-associated proteins in two independent mRNA-bound proteome studies using human hepatocytic HuH-7 cells and human embryonic kidney HEK293 cells (69, 70). Interestingly, human PRMT1 localizes to cytoplasmic granules upon arsenite treatment, suggesting further similarities between the two enzymes (71). Neither *Tb*PRMT1 nor *Hs*PRMT1 contains a known RNA binding domain. We identified specific residues in *Tb*PRMT1 that, when mutated, attenuate its RNA binding activity, suggesting it may be possible to create a nucleic-acid-binding-deficient mutant that could in turn be used to further probe the biological significance of this feature.

In summary, we demonstrate here that *Tb*PRMT1 affects T. brucei virulence, metabolism, and ability to cope with starvation stress. We identified a subset of proteins that change their mRNA association upon *Tb*PRMT1 depletion, providing new directions for examining the role played by arginine methylation in regulation of RNA binding proteins. Furthermore, we established that *Tb*PRMT1 itself has the ability to associate with nucleic acids, a finding that may have more widespread implications among PRMT1 homologs. We identified candidate *Tb*PRMT1 targets that play a role in a broad spectrum of processes, extending the impact of this work to numerous fields of study, such as life cycle progression, translation, stress response, and metabolism regulation. Future studies will be aimed at distinguishing primary effects of *Tb*PRMT1 loss from downstream proteome changes and deciphering the role that nucleic acid binding plays in *Tb*PRMT1’s mechanism of action.

## MATERIALS AND METHODS

For details including buffer compositions and antibody sources, please refer to Text S1 in the supplemental material.

10.1128/mBio.02430-18.1 TEXT S1 Detailed experimental procedures. Download Text S1, DOCX file, 0.05 MB. This is a work of the U.S. Government and is not subject to copyright protection in the United States. Foreign copyrights may apply.

### T. brucei cell culture and cell lines.

PF T. brucei strain 29-13 and all cell lines derived from this strain were grown in SM medium supplemented with 10% FBS. The BF SM427 strain was cultured in HMI-11 medium supplemented with 10% FBS. The ENZ KO cell line was created by replacing both ENZ alleles with drug resistance markers. The PRO-MHT cell line contains a tetracycline-inducible C-terminally Myc-His-TAP-tagged PRO construct derived from pLEW100. The PRO-mNG-TY cell line was created using pPOTv6-mNG according to the published protocol (72). The ENZ RNAi cell line was created as described in reference 11.

### Statistical analysis.

Statistical significance of doubling time differences was calculated using multiple-comparison one-way ANOVA (Fig. 1). Western blot differences were tested by Student's *t* test (Fig. 2 and 3). GO term enrichment *P* values were calculated by Fisher’s exact test with Bonferroni correction (Fig. 4A). *P* value of overlap of two protein data sets was determined by R p-hyper function (Fig. 4B). Significance of differences between amounts of granule-containing cells in uninduced and induced ENZ RNAi cell lines was determined by the chi-square test (Fig. 5). For proteomics data sets, statistical significance between groups was evaluated using a Student *t* test (Table S1 and S2).

### Mouse infection assay.

Fourteen female CD-1 mice (10 to 12 weeks; Charles River) were infected by intraperitoneal injection with 1 × 10^5^ bloodstream-form T. brucei parasites; half were *Tb*PRMT1 ENZ knockout (ENZ KO; *n* = 7) or SM parental strain (WT; *n* = 7). The course of infection was monitored, and time to death was recorded. Experiments were carried out in accordance with protocols approved by the Institutional Animal Care and Use Committee (IACUC) of Clemson University.

### Whole-cell proteome and mRNA-bound proteome.

Approximately 4 × 10^6^ cells were harvested, and whole-cell proteomes were quantified as described in Text S1. mRNA-bound proteome purification was performed as described previously, with only minor modifications (30). The protein description in Table 2 includes posttranscriptional activator/repressor annotation that was established by a genome-wide tethering screen (53).

### Immunolocalization and fluorescence *in situ* hybridization.

NOPP44/46-1 was immunolocalized using antibody against the protein (1:2,000 dilution). FISH was performed using an oligo(dT) probe fused to Alexa Fluor 488 in an overnight (O/N) hybridization protocol.

### *In vitro* RNA binding, competition, and EMSA.

A 102-nt RNA fragment of pBluescript SK− plasmid sequence was *in vitro* transcribed. A UV-cross-linking-based RNA binding assay was performed as described previously with minor modifications (73). PRMT molarity was determined per dimer in all cases. Affinity for different polynucleotides was determined by adding a mass excess of polynucleotide into the reaction mixture prior to the addition of the protein. To perform EMSA, a 47-nt RNA fragment of the pBluescript SK− plasmid was transcribed and incubated with a recombinant protein. Reaction mixtures were resolved on native PAGE.

10.1128/mBio.02430-18.5 TABLE S3 Proteins associated with PRO-MHT in PF T. brucei were identified. Tab “All proteins PRO-MHT” contains entire data set. For tab “Refined PRO-MHT set,” data set was refined by eliminating all proteins that were identified by <2.5× peptides over the negative control, using the replicate with lower peptide count for that specific protein. Download Table S3, XLSX file, 5.3 MB. This is a work of the U.S. Government and is not subject to copyright protection in the United States. Foreign copyrights may apply.

10.1128/mBio.02430-18.6 TABLE S4 List of primers used in this study. Download Table S4, XLSX file, 0.01 MB. This is a work of the U.S. Government and is not subject to copyright protection in the United States. Foreign copyrights may apply.
