# Supplementary material for: Trypanosoma brucei PRMT1 Is a Nucleic Acid Binding Protein with a Role in Energy Metabolism and the Starvation Stress Response
Source: mBio. 2018 Dec 18;9(6):e02430-18. doi: 10.1128/mBio.02430-18 (PMC6299225; doi:10.1128/mBio.02430-18)
Supplement: FIG S1 [file mbo006184221sf1.pdf]

Figure S1

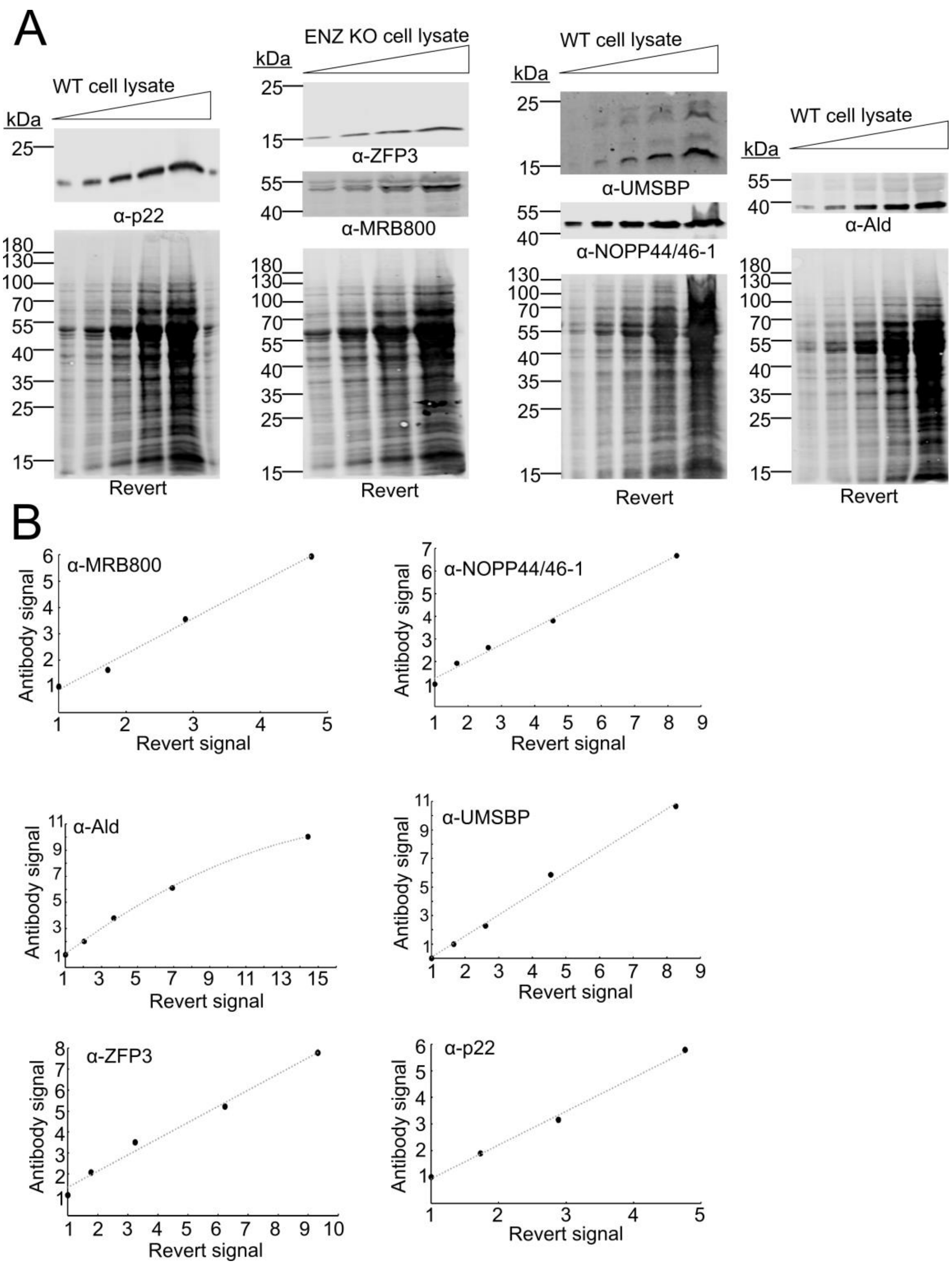

A
WT cell lysate
kDa
25
α-p22
180
130
100
70
55
40
35
25
15
Revert
ENZ KO cell lysate
kDa
25
15
α-ZFP3
55
40
α-MRB800
180
130
100
70
55
40
35
25
15
Revert
WT cell lysate
kDa
25
15
α-UMSBP
55
40
α-NOPP44/46-1
130
100
70
55
40
35
25
15
Revert
WT cell lysate
kDa
55
40
α-Ald
180
130
100
70
55
40
35
25
15
Revert
B
α-MRB800
Antibody signal
Revert signal
α-NOPP44/46-1
Antibody signal
Revert signal
α-Ald
Antibody signal
Revert signal
α-UMSBP
Antibody signal
Revert signal
α-ZFP3
Antibody signal
Revert signal
α-p22
Antibody signal
Revert signal

Fig. S1 *Determination of antibody linear range*. Increasing amounts of BF *T. brucei* cell lysate were probed with indicated antibodies. Signal of both antibody and Revert (LC) were normalized to the signal in the first lane and plotted. Trendline was plotted and equation of the trendline was used to determine values in Fig. 2*A*.
