# Supplementary material for: Trypanosoma brucei PRMT1 Is a Nucleic Acid Binding Protein with a Role in Energy Metabolism and the Starvation Stress Response
Source: mBio. 2018 Dec 18;9(6):e02430-18. doi: 10.1128/mBio.02430-18 (PMC6299225; doi:10.1128/mBio.02430-18)
Supplement: TEXT S1 [file mbo006184221s1.docx]

DETAILED EXPERIMENTAL PROCEDURES

*T. brucei cell culture, growth curves and generation of cell lines and antibodies –* PF 29-13 strain (1) was cultured in SM media (Hyclone) supplemented with MEM vitamin solution (Gibco), penicillin, streptomycin and 10% FBS. BF Single Marker 427 strain (information about the identity and genealogy of this strain is available <http://tryps.rockefeller.edu/DocumentsGlobal/lineage_Lister427.pdf>) was cultured in HMI-11 media supplemented with 10% FBS (2). All electroporations were performed using an Amexa Nucleofactor system.

ENZ KO was created by replacement of both alleles with resistance cassettes. All primer sequences are available in Table S4. pJET1.2 (Fermentas) vector was digested with XhoI/XbaI and ligated back together with phosphorylated primer pairs MCS 4A, MCS 4B and MCS 5A, MCS 5B to create pKOJET. Next, resistance cassettes puromycin (PURO) and blasticidin (BSD), were PCR amplified, digested with BamHI/KpnI, and ligated into the pKOJET vector digested with BamHI/KpnI. We then amplified 125 nt of the ENZ 5’ UTR and 3’UTR sequences, and cloned them to flank the resistence gene. Primers ENZ 5'UTR AMP and ENZ 3'UTR AMP were then used to amplify the UTR flanked resistance gene. Five μug of each phenol-chloroform purified PCR was then used for two consequent transfections. First, the PURO construct was transfected, transfectants were selected using 50 ng/ml puromycin, and clones were obtained by limiting dilution. Incorporation of the construct was checked by PCR on genomic DNA (gDNA) using primers ENZ 5'UTR AMP and ENZ 3'UTR AMP. A single positive clone was selected and transfected with BSD cassette PCR in the absence of selection drugs. Transformants were then selected by adding 5 μg/ml blasticidin and 50 ng/ml puromycin. Clones were obtained by limiting dilution and screened for incorporation of both cassettes by ENZ 5'UTR AMP and ENZ 3'UTR AMP primer PCR on gDNA. The absence of ENZ ORF was confirmed by gDNA PCR using PRO-MHT 5’ and PRO-MHT 3’ primers**.** To express untagged addback, the ENZ ORF including stop codon was cloned into pLEW100 plasmid carrying phleomycin resistance. The resulting plasmid was NotI linearized, and transfectants were selected with 2.5 μg/ml phleomycin. Expression was induced with 4 μg/ml doxycycline.

PRO-MHT construct was created by amplifying the PRO ORF using PRO-MHT 5’ and PRO-MHT 3’ primers and cloning into pLew100 plasmid harboring a C-terminal Myc-His-TAP (MHT) at the HindIII/XhoI cut sites. Plasmid was linearized by NotI digestion and transfected into PF 29-13 strain. Transformants were selected with 1 μg/ml of puromycin, and clones were obtained by limiting dilution.

PRO-mNG-TY cell line was created by *in situ* addition of C-terminal Neon Green-Ty1 (mNG) tag. Primers 7CTb927.10.3560F and 7CTb927.10.3560R were used to amplify the tag, including the resistance marker, from pPOTv6-mNG according to the published protocol (3). The PCR amplicons was purified and transfected, and transformants were selected with 0.1 μg/ml of puromycin. Clones were obtained by dilution as recommended in published protocol (3).

ENZ or PRO doxycycline inducible RNAi cell lines were generated as described in (4). Briefly, plasmids were linearized using the NotI restriction site and electroporated into PF cells. Transformants were selected with 2.5 µg/ml phleomycin and clones were obtained by limiting dilution. RNAi was induced for four days with 4 µg/ml of doxycycline.

ENZ and PRO antibodies (ABs) were generated against peptide CHVDFEGALQISHYDQFRLR or CIETKGTYNYQRY, respectively, by Bethyl Laboratories. MRB800 (Tb927.7.800) antibody was generated against maltose binding protein (MBP)-taggeombinant protein by Bethyl laboratories. p22 antibody and DRBD18 antibody was raised as previously described (5, 6). ZFP3 antibody was generated against peptide DSSQMQQVGHDVPPMA (Bethyl laboratories). NOPP44/46-1 peptide antibody was gifted by Dr. Noreen Williams (7), UMSBP antibody was donated by Dr. Joseph Shlomai (8), glycosomal aldolase antibody was a kind gift from Dr. Meredith Morris (9), Dr. Keith Matthews provided us with PAD2 antibody (10), and HSP70 antibody was a gift from Dr. Jay Bangs. ASYM24 (ADMA antibody) was purchased from EMD Millipore. MeR4 (MMA antibody) was purchased from Cell Signaling (Danvers, MA, USA). TY antibody was purchased from UAB Hybridoma Facility, Birmingham, AL.

Growth curves were obtained by counting cumulative cell numbers every day. Fig. 1B was generated using two clones, each in duplicate. Fig. 1C used single measurement of two clones for three consecutive days. Doubling times were calculated using Prism software. Statistical analysis used 1 way ANOVA multiple comparisons.

*Western blotting and quantitative western blotting –* All western blotting was performed using nitrocellulose membranes, BioRad Trans-Blot® Turbo™, LiCor fluorescent secondary antibodies and LiCor Odyssey CLx imaging system. Blots were blocked for 20 min at RT and incubated O/N at 4°C with primary antibody. Blocking solution used corresponded with the solution used for primary antibody dilution. When the primary antibody was diluted in TBS-0.05% Tween (TBS-T) with milk, blocking was done using 5% milk in TBS. ENZ antibody was used at 1:1000 in Odyssey® Blocking Buffer (PBS based). ASYM24 was used at 1:500 in Odyssey® Blocking Buffer (Casein based). MeR4 was diluted at 1:1000 in 2% milk in TBS-T. HSP70 antibody was used at 1:2000 in 2% milk in TBS-T. MRB800 antibody was used at 1:1000 in 1% milk. p22 antibody was used at 1:5000 in 2% milk in TBS-T. ZFP3 antibody was used at 1:1000 in 1% milk in TBS-T. UMSBP antibody was used at 1:5000 in 3% milk TBS-T. NOPP44/46-1 antibody was used at 1:5000 in 2% milk in TBS-T. Aldolase antibody was used at 1:5000 in 2% milk in TBS-T.

To determine the linear range of antibody protein recognition, increasing amounts of cell lysate were loaded on the same gel as the sample that was to be quantified. Membranes were stained with whole-protein stain Revert (LiCor) and both antibody signal and Revert signal were quantified. The antibody signal and corresponding revert signal were graphed and trendlines were determined using the least squares method. The equation of the trendline was used to calculate expected signal for test samples based on their Revert signal. The actual signal was divided by expected signal. Values were normalized to the WT sample average. Statistical significance was determined by Student’s t-test. For mRNA bound proteome samples, three biological replicates of each WT and KO experiment were analyzed by western blot. Antibody signal was normalized to Revert signal and mean value was plotted. The signal quantification values corresponded to values within linear range determined in whole cell proteome verification. Statistical significance was determined by Student’s t-test.

*Mouse infection assay –* For mouse infections, *Tb*PRMT1 ENZ knock-out (ENZ KO) or SM parental strain (WT) *T. brucei* were re-selected for one week in HMI-11 media supplemented with 5 µg/ml blasticidin and 50 ng/ml puromycin, followed by one week in media without antibiotics, and knock-out genotype was confirmed by gDNA PCR. For infection, parasite cultures were washed 1x and resuspended in sterile 1X PBS at a concentration of 5 x 10^5^ cells/ml (inoculum volume of 200 µl). Fourteen female CD-1 mice (10-12 weeks; Charles River) were infected by intraperitoneal injection with 1 x 10^5^ bloodstream form *T. brucei* parasites, half were *Tb*PRMT1 ENZ knock-out (ENZ KO; n=7) or SM parental strain (WT; n=7). Course of infection was monitored and time to death was recorded. Parasitemia was monitored periodically by microscopic examination of blood smears from tail blood. Mice were monitored daily for general appearance, behavior, and weight loss. If a mouse reached a humane endpoint (parasitemia ≥ 10^8^, >20% weight loss, or obvious distress), then the mouse was euthanized and time of death marked as the following day. Significance of difference in average time-to-death between mice infected with ENZ KO and parental WT control was assessed by one-tailed Student's t-test. Experiments were carried out in accordance with protocols approved by the Institutional Animal Care and Use Committee (IACUC) of Clemson University.

*Whole cell quantitative proteomics and mRNA bound proteome purification –* To quantify proteins present in WT and ENZ KO cell line, WT and ENZ KO cell lines were harvested at a cell density <10^6^ cells/ml. Five replicates for each cell line were washed 3x with PBS-6 mM glucose (PBS-G) and pelleted. Pellets containing ~4x10^6^ cells were analyzed by quantitative mass spectrometry as described below.

To identify mRNA bound proteins in BF *T. brucei*, 1x10^9^ WT or ENZ KO cells were harvested. Cells were washed with PBS-G, re-suspended in 4 ml PBS-G, divided onto four wells of 24-well culture plate, placed 2 cm from the UV bulbs and cross-linked at 200 mJ using UV Stratalinker 2400 (Stratagene). Cells were pelleted and flash-frozen. Each pellet containing 5x10^8^ cells was re-suspended in 1.5 ml lysis buffer (20 mM Tris-HCl (pH 7.5), 500 mM LiCl, 0.5% LiDS, 1 mM EDTA, 5 mM DTT, cOmplete™ Protease Inhibitor Cocktail Tablet (Roche) 1 tbl/ 200 ml) and homogenized by passing through 27-gauge needle ten times. Five hundred µl of oligo-dT magnetic beads (NEB) was added and incubated with the lysate for 1 hour at 4°C while rocking. Lysate was saved, beads were washed 3x with lysis buffer, 3x with wash buffer (20 mM Tris pH 7.5, 140 mM LiCl, 1 mM EDTA, 0.5% NP40, 0.5 mM DTT) and 1x with 10 mM Tris-HCl (pH 7.5). Samples were eluted in 30 µl 10 mM Tris-HCl (pH 7.5) by heating to 80°C for 2 min. Beads were added back to lysate and whole procedure was repeated two times. Elutions were pooled for total of 180 µl/1x10^9^ starting cell amount. Samples were analyzed by mass spectrometry as described below. To confirm the results by WB, quarter scale experiment was repeated in triplicate.

To confirm PRO *in vivo* mRNA association, PF PRO-MHT cells were induced for 4 days with 4 µg/ml of doxycycline. Cells (9x10^9^) were harvested, re-suspended in 16 ml PBS-G and half were UV cross-linked (7 x 200 mJ, 6-well plate, 2.75 ml/well, 3 cm from lamp). Cells were pelleted and flash frozen. Each pellet of 4.5x10^9^ cells was re-suspended in 2.5 ml lysis buffer (see above). Lysate passed 15x through 26-gauge needle and spud down at 700 x g for 3 min to clear. Fifty μl of oligo-dT magnetic beads (NEB) were added, and samples were incubated and washed as described above. Twenty-five μl of 10 mM Tris-HCl (pH 7.5) was used for each elution for total of 75 ul/sample. Four μg RNAse A/ sample was added, and reactions were incubated for 30 min at 37°C before running on SDS-PPAGE.

PRO *complex affinity purification –* The PRO-MHT cell line was either left uninduced or induced for 2 days with 4 µg/ml doxycycline. Cells (1x10^10^) were harvested and resuspended in 9 ml IPP150 buffer (10 mM Tris (pH 8), 150 mM NaCl, 0.1% NP40) containing 1mM PMSF, 1 tbl/100 ml cOmplete™ Protease Inhibitor Cocktail Tablet (Roche), 4 tbl/100 ml PhosSTOP™ phosphatase inhibitors (Roche) and 2 mM activated Na_3_VO_4_. Cells were lysed by 1% Triton-X for 20 min on ice. Lysate was cleared by centrifugation and incubated with 200 µl of IgG Sepharose 6 Fast Flow affinity resin (GE Healthcare) for 2.5 hours at 4°C. Beads were washed with IPP150 buffer and TEVCB buffer (10 mM Tris-HCl (pH 8.0), 150 mM NaCl, 0.1% NP40, 0.5 mM EDTA, 1 mM DTT). 1 ml TEVCB buffer and 10 µl of AcTEV protease (Thermo Fisher Scientific) was added to beads and incubated for 1 hour at 28°C and then at 4°C O/N. TEV elution was collected and analyzed by LC-MS/MS as described below**.** GO term analysis was performed using the TriTryp database. Values depicted are p-values adjusted by Bonferroni correction. Overlap with the stress granule proteome was determined as follows. Population size was set to the number of all proteins identified in Fritz *et al.* (11). Sample size was set to a cross of proteins identified in our PRO purification and proteins identified in Fritz *et. al.* p-hyper R function was used to determine p-value of overlap between proteins overrepresented in stress granules and PRO purification.

*Protein Extraction and Precipitation/ On-Pellet Digestion – T. brucei* cell pellets were lysed in ice-cold lysis buffer (50 mM Tris-formic acid, 150 mM NaCl, 0.5% sodium deoxycholate, 1% SDS, 2% NP-40, pH 8.0) with protease inhibitor (Complete, Mini, EDTA-free; Roche, Mannheim, Germany). The cell lysate was sonicated on ice for brief intervals (Qsonica, Newtown, CT, USA) and centrifuged at 18,000 × g for 30 min at 4 °C. The supernatant was transferred and the protein concentration was determined by the bicinchoninic acid (BCA) assay (Thermo Fisher Scientific, San Jose, CA). Samples containing 100 μg protein were reduced by the addition of 5 mM dithiothreitol (DTT) for 30 minutes, followed by alkylation using 40 mM iodoacetamide for 30 minutes at 37 °C in darkness. The protein mixture was precipitated by stepwise addition of 5 volumes of cold acetone with continuous vortex and then incubated at -20°C overnight. Centrifugation at 18,000 g for 30 min at 4 °C was performed. The pellets were washed using an 85/15% (v/v) chilled acetone/water solution, and then partially air-dried. The pellets were resuspended with 100 μl Tris-formic acid buffer (50 mM Tris, pH 8.5) and digested using trypsin at a 1:20 (w/w) enzyme/substrate ratio. The mixture was incubated at 37 °C overnight with constant mixing at 200 RPM. A 1% (v/v) final concentration of formic acid was added to terminate digestion. For co-IP eluates, the same precipitation/on-pellet digestion method was used.

*Nano-LC-MS/MS analysis* *–* A Thermo Scientific UltiMate 3000 RSLCnano system and an Orbitrap Fusion Lumos mass spectrometry (Thermo Fisher Scientific, San Jose, CA) were employed. The mobile phase A contains 2% acetonitrile in 0.1% formic acid and mobile phase B contains 88% acetonitrile in in 0.1% formic acid. The nano-LC column was heated at 52°C. Four µl of digested mixtures were loaded onto a large-ID trap (300 µm ID x0.5 cm, packed with Zorbax 3-µm C18 material) with 1% B at a flow rate of 10 µL/min for 3 min. The trapped peptides were then back-flushed onto the nano-LC column (75 µm ID x 60 cm, packed with Waters XSelect CSH 2.5-µm C18 material) at a flow rate of 250 nL/min. A 180-min gradient was used and the optimized gradient profile was as following: 4% over 3 min; 4 to 11% over 5 min; 11 to 28% over 117 min; 28 to 50% over 10 min; 50 to 97% over 1 min and isocratic for 17min; and finally isocratic at 4% of B for 27 min.

Mass spectrometry data was acquired under data-dependent product ion scan mode with a cycle time of 3s. One scan cycle included a survey scan (m/z 400-1500) at a resolution of 120,000 with an AGC target of 5×10^5^ and a maximum injection time of 50 ms. MS2 was performed by isolation at 1.2 Th with the quadrupole for high energy collision dissociation (HCD) fragmentation and detected by Orbitrap at a resolution of 15,000 with an AGC target of 5×104. The maximum injection time was 50 ms, the normalized activation energy was 30% with a 5% of stepped collision energy, and the activation q was 0.25. Dynamic exclusion was enabled with repeat count of 1 and exclusion duration of 45 s.

*Protein identification and quantification* *–* The mass spectrometry raw files were searched against the *T. brucei* protein database (released on March, 2017 at www.TriTrypDB.org) with a total of 11,202 protein entries using the Proteome Discoverer v1.4 (Thermo Fisher Scientific). The search parameters were set as follows: 10 ppm tolerance for precursor ion mass and 0.02 Da for fragment ion mass with fully tryptic peptides restraint and a maximum of two missed cleavages. Static carbamidomethylation of cysteine and dynamic oxidation of methionine, phosphorylation of serine, tyrosine and threonine, mono/di/tri-methylation of lysine and arginine, and n-terminal acetylation were used. The false discovery rate was detected by using a target-decoy search strategy. Scaffold 4.5 (Proteome Software, Portland, OR) was used to validate MS/MS-based peptide and protein identification. The false discovery rates of 0.1% at peptide level and 1% at protein level were applied in this study. An ion current-based quantification method (IonStar processing pipeline) was described previously (12, 13). The quantitative frames were determined based on m/z (width: 5 ppm) and retention time (width: 1 min). Peptides or frames shared among different protein groups were excluded from quantitative analysis. The ion current intensities of each protein were normalized by the LOESS method. Intensities for frames with the same sequence were combined to be the unique peptide intensity. Then intensities for unique peptides of the same protein were further combined to be the protein intensity with Grubbs’ test (a minimum dataset presence 2 and p-value cutoff of 0.01). The relative expression ratio was calculated by the average ion-current intensities of five replicates in each group. Statistical significance between groups was evaluated using a Student’s t-test. Thresholds of 1.5-fold change and a p-value cutoff of 0.05 were used to define the altered proteins. This statistical evaluation was previously shown better sensitivity with a publicly acceptable FDR when compared to evaluation using multiple hypothesis testing correction (Sequential Bonferroni test, Benjamini and Hochberg test, Sequential Fisher combined probability test) (12).

*Immunolocalization and Fluorescence In Situ Hybridization –* To determine subcellular localization of *Tb*PRMT1 and NOPP44/46-1, PRO-nGreen-TY cells or BF single marker cells were fixed in 2-4% formaldehyde in PBS, permeabilized (0.5% NP40, PBS), blocked (PBS, 10% normal rabbit serum (NRS), 0.1% NP40), and incubated with 1:500 α-TY antibody or 1:2000 α-NOPP44/46-1 in block solution for 1 hour. Samples were washed 3x (1% NRS, PBS), incubated with either 1:400 Alexa 488 goat anti-mouse (Invitrogen) or Alexa Fluor 594 goat anti-rabbit (Invitrogen) for 30 min, washed 3x (1% NRS, PBS), and mounted using ProLong Gold Antifade Mountant with DAPI (Invitrogen). Images were collected as described previously (14). Image Z stacks (0.2 μm Z‐increment) were collected with capture times from 50 to 500 milliseconds (100x PlanApo, oil immersion, 1.46 na) on a Zeiss Axioimager M2 stand equipped with a rear‐mounted excitation filter wheel, a DAPI/FITC/Texas Red emission cube, differential interference contrast optics, and an Orca ER CCD camera (Hamamatsu). All images were collected with Volocity 6.1 Acquisition Module (Improvision Inc.), and individual Z stacks were deconvolved by a constrained iterative algorithm, pseudocolored if needed, and merged using Volocity 6.1 Restoration Module. All images presented are summed stack projections of merged channels.

To visualize mRNA in *Tb*PRMT1^ENZ^ RNAi cell line, knock-down was induced for three days by addition of 4 μg/ml of doxycycline. Cells were pelleted to normalize the concentration and transferred to either media or PBS. Cells were incubated for two hours at 27°C, fixed in 4% formaldehyde in PBS, and adhered to poly-lysine coated slides. Cells were incubated with 25 mM NH_4_Cl and permeabilized and blocked by incubation with 0.5% saponin and 2% BSA. Cells were then incubated with pre-hyb solution (2% BSA, 5xDenhard’s solution, 4xSSC, 5% Dextran sulfate, 35% deionized formamide, 0.5 μg/μl Torula yeast RNA, 10 U/ml RNAse inhibitor) and incubated with 2 ng/μl oligo d(T) probe fused to Alexa fluor 488 in pre-hyb solution O/N. Slides were washed, and images were acquired as described above. Cells were classified as normal, intermediate or granule-containing. 300 - 500 cells were counted in each biological replicate. P-values were calculated using Chi-Square Test.

*Recombinant protein expression, in vitro RNA binding, competition and EMSA –* Recombinant proteins were generated as described in (15-17). To determine *in vitro* RNA binding properties, primers “All pBSC” and “pBSC-A20” were used to amplify a 102 nt fragment of pBluescript SK- plasmid. The PCR was used as a template to generate ^32^P UTP (800 Ci/mmol) bodylabeled RNA using the MAXIscript™ T7 Transcription Kit (Invitrogen). RNA was gel purified and incubated with recombinant proteins in the following 20 μl reaction: 0.5 nM RNA, 0.75 μM protein dimers, 4.5 mM HEPES (pH 7.5), 1.6 mM MgCl_2_, 0.375 mM DTT, 1.13 mM ATP, 3.75 mM creatine phosphate, 75 μM EDTA, 7.5 μg/ml Torula yeast RNA, 4.5% glycerol, 0.5 μl RNAse inhibitor. Complexes were UV crosslinked (10 min, 3 cm from lamp, UV Stratalinker 2400), treated with 30 μg RNAse A for 15 min at 37°C, resolved on SDS-PAGE, Coomassie stained, gels were dried, and signal was visualized using a phosphoimager screen. To determine affinity for polynucleotides, pBluescript was linearized with Hind III and used as a template in a MAXIscript™ T7 Transcription Kit reaction containing ^32^P UTP (800 Ci/mmol). The resulting 47 nt product was gel purified and reactions were setup as above. The shorter fragment was chosen to eliminate possible role of poly(A) tail present in the 102 nt construct. In addition, each reaction contained indicated mass excess of polynucleotides (Sigma). To determine *Tb*PRMT1 preference for RNA/DNA, unlabeled RNA was synthesized using HindIII linearized pBluescript plasmid in a MEGAscript T7 Transcription Kit (Invitrogen). A DNA oligonucleotide of corresponding sequence was purchased (Invitrogen). Reactions were set up and treated as above, except the protein was present in 75 nM concentration. Competitor was present in mass excess as indicated. To perform EMSA, pBluescript plasmid was linearized with HindIII and 47 nt RNA was transcribed using MAXIscript™ T7 Transcription Kit reaction containing ^32^P UTP (800 Ci/mmol). RNA was gel purified and incubated with recombinant protein in a following reaction: 2.1 mM MgCl_2_, 0.5 mM DTT, 0.1 mM EDTA, 6% glycerol, 0.01% NP-40, 0.1 mg/ml Torula yeast RNA, 5 μg/ml heparin, 500 pM labelled RNA, indicated molar amount of PRMT dimers. Reactions were resolved on native PAGE, gels were dried and exposed to phosphorimager screen.

*Whole cell nascent protein [^35^S]Met labelling –* To assay translation efficiency changes between WT and ENZ KO BF cell lines, 5x10^6^ cells were harvested, washed with PBS-G, re-suspended in media lacking methionine and incubated at 37°C for 15 min. Five μl of [^35^S]Met (10.2 mCi/ml, Perkin Elmer)/ sample was added and incubated for 1 hour at 37°C. Cells were washed with PBS-G, re-suspended in 30 μl 1x SDS loading dye and proteins were separated on SDS PAGE. Coomassie stained gels were dried and imaged by phophorimaging.

1. Wirtz E, Leal S, Ochatt C, Cross GA. A tightly regulated inducible expression system for conditional gene knock-outs and dominant-negative genetics in Trypanosoma brucei. Molecular and biochemical parasitology. 1999;99(1):89-101. PubMed PMID: 10215027.

2. Hirumi H, Hirumi K. Continuous cultivation of Trypanosoma brucei blood stream forms in a medium containing a low concentration of serum protein without feeder cell layers. J Parasitol. 1989;75(6):985-9. PubMed PMID: 2614608.

3. Dean S, Sunter J, Wheeler RJ, Hodkinson I, Gluenz E, Gull K. A toolkit enabling efficient, scalable and reproducible gene tagging in trypanosomatids. Open biology. 2015;5(1):140197. Epub 2015/01/09. doi: 10.1098/rsob.140197. PubMed PMID: 25567099; PubMed Central PMCID: PMCPMC4313374.

4. Lott K, Zhu L, Fisk JC, Tomasello DL, Read LK. Functional interplay between protein arginine methyltransferases in Trypanosoma brucei. MicrobiologyOpen. 2014;3(5):595-609. Epub 2014/07/22. doi: 10.1002/mbo3.191. PubMed PMID: 25044453.

5. Sprehe M, Fisk JC, McEvoy SM, Read LK, Schumacher MA. Structure of the Trypanosoma brucei p22 protein, a cytochrome oxidase subunit II-specific RNA-editing accessory factor. The Journal of biological chemistry. 2010;285(24):18899-908. Epub 2010/04/16. doi: M109.066597 [pii]

10.1074/jbc.M109.066597. PubMed PMID: 20392699; PubMed Central PMCID: PMC2881812.

6. Lott K, Mukhopadhyay S, Li J, Wang J, Yao J, Sun Y, et al. Arginine methylation of DRBD18 differentially impacts its opposing effects on the trypanosome transcriptome. Nucleic acids research. 2015. Epub 2015/05/06. doi: 10.1093/nar/gkv428. PubMed PMID: 25940618.

7. Hellman K, Prohaska K, Williams N. Trypanosoma brucei RNA binding proteins p34 and p37 mediate NOPP44/46 cellular localization via the exportin 1 nuclear export pathway. Eukaryotic cell. 2007;6(12):2206-13. Epub 2007/10/09. doi: 10.1128/ec.00176-07. PubMed PMID: 17921352; PubMed Central PMCID: PMCPmc2168238.

8. Onn I, Kapeller I, Abu-Elneel K, Shlomai J. Binding of the universal minicircle sequence binding protein at the kinetoplast DNA replication origin. The Journal of biological chemistry. 2006;281(49):37468-76. Epub 2006/10/19. doi: 10.1074/jbc.M606374200. PubMed PMID: 17046830.

9. Bauer ST, McQueeney KE, Patel T, Morris MT. Localization of a Trypanosome Peroxin to the Endoplasmic Reticulum. The Journal of eukaryotic microbiology. 2017;64(1):97-105. Epub 2016/06/25. doi: 10.1111/jeu.12343. PubMed PMID: 27339640; PubMed Central PMCID: PMCPMC5215699.

10. Dean S, Marchetti R, Kirk K, Matthews KR. A surface transporter family conveys the trypanosome differentiation signal. Nature. 2009;459(7244):213-7. Epub 2009/05/16. doi: 10.1038/nature07997. PubMed PMID: 19444208; PubMed Central PMCID: PMCPMC2685892.

11. Fritz M, Vanselow J, Sauer N, Lamer S, Goos C, Siegel TN, et al. Novel insights into RNP granules by employing the trypanosome's microtubule skeleton as a molecular sieve. Nucleic acids research. 2015;43(16):8013-32. Epub 2015/07/19. doi: 10.1093/nar/gkv731. PubMed PMID: 26187993; PubMed Central PMCID: PMCPMC4652759.

12. Tu C, Sheng Q, Li J, Shen X, Zhang M, Shyr Y, et al. ICan: an optimized ion-current-based quantification procedure with enhanced quantitative accuracy and sensitivity in biomarker discovery. Journal of proteome research. 2014;13(12):5888-97. Epub 2014/10/07. doi: 10.1021/pr5008224. PubMed PMID: 25285707; PubMed Central PMCID: PMCPmc4261937.

13. Shen X, Shen S, Li J, Hu Q, Nie L, Tu C, et al. An IonStar Experimental Strategy for MS1 Ion Current-Based Quantification Using Ultrahigh-Field Orbitrap: Reproducible, In-Depth, and Accurate Protein Measurement in Large Cohorts. Journal of proteome research. 2017;16(7):2445-56. Epub 2017/04/18. doi: 10.1021/acs.jproteome.7b00061. PubMed PMID: 28412812; PubMed Central PMCID: PMCPMC5914162.

14. Umaer K, Bush PJ, Bangs JD. Rab11 mediates selective recycling and endocytic trafficking in Trypanosoma brucei. Traffic. 2018;19(6):406-20. Epub 2018/03/28. doi: 10.1111/tra.12565. PubMed PMID: 29582527.

15. Kafkova L, Debler EW, Fisk JC, Jain K, Clarke SG, Read LK. The Major Protein Arginine Methyltransferase in Trypanosoma brucei Functions as an Enzyme-Prozyme Complex. The Journal of biological chemistry. 2017;292(6):2089-100. doi: 10.1074/jbc.M116.757112. PubMed PMID: 27998975; PubMed Central PMCID: PMC5313084.

16. Fisk JC, Sayegh J, Zurita-Lopez C, Menon S, Presnyak V, Clarke SG, et al. A type III protein arginine methyltransferase from the protozoan parasite Trypanosoma brucei. The Journal of biological chemistry. 2009;284(17):11590-600. PubMed PMID: 19254949.

17. Fisk JC, Zurita-Lopez C, Sayegh J, Tomasello DL, Clarke SG, Read LK. TbPRMT6 is a type I protein arginine methyltransferase that contributes to cytokinesis in Trypanosoma brucei. Eukaryotic cell. 2010;9(6):866-77. Epub 2010/04/27. doi: 10.1128/ec.00018-10. PubMed PMID: 20418380; PubMed Central PMCID: PMCPmc2901642.
